# Organometallic Half-Sandwich Complexes of 8-Hydroxyquinoline-Derived Mannich Bases with Enhanced Solubility: Targeting Multidrug Resistant Cancer

**DOI:** 10.1021/acs.inorgchem.4c04398

**Published:** 2024-12-05

**Authors:** Tamás Pivarcsik, Szilárd Tóth, Szonja P. Pósa, Nóra V. May, Éva Kováts, Gabriella Spengler, Izolda Kántor, Alexandra Rolya, Tivadar Feczkó, István Szatmári, Gergely Szakács, Éva A. Enyedy

**Affiliations:** † MTA-SZTE Lendület Functional Metal Complexes Research Group, University of Szeged, Dóm tér 7-8, Szeged H-6720 , Hungary; ‡ Department of Molecular and Analytical Chemistry, Interdisciplinary Excellence Centre, University of Szeged, Dóm tér 7-8, Szeged H-6720, Hungary; § Drug Resistance Research Group, Institute of Molecular Life Sciences, HUN-REN Research Centre for Natural Sciences, Magyar Tudósok krt. 2, Budapest H-1117, Hungary; ∥ National Laboratory for Drug Research and Development, Magyar Tudósok krt. 2, Budapest H-1117 , Hungary; ⊥ Centre for Structural Science, HUN-REN Research Centre for Natural Sciences, Magyar Tudósok krt. 2, Budapest H-1117, Hungary; # Institute for Solid State Physics and Optics, HUN-REN Wigner Research Centre for Physics, P.O. Box 49, Budapest H-1525, Hungary; ∇ Department of Medical Microbiology, Albert Szent-Györgyi Health Center and Albert Szent-Györgyi Medical School, University of Szeged, Semmelweis u. 6, Szeged H-6725, Hungary; ○ Institute of Materials and Environmental Chemistry, HUN-REN Research Centre for Natural Sciences, Magyar Tudósok krt. 2, Budapest H-1117, Hungary; ◆ Faculty of Engineering, University of Pannonia, Egyetem u. 10, Veszprém H-8200, Hungary; ¶ Institute of Pharmaceutical Chemistry, HUN-REN-SZTE Stereochemistry Research Group, University of Szeged, Eötvös u. 6, Szeged H-6720, Hungary; ⋈ Center for Cancer Research, Medical University of Vienna, Borschkegasse 8a, Wien, Vienna A-1090 Austria

## Abstract

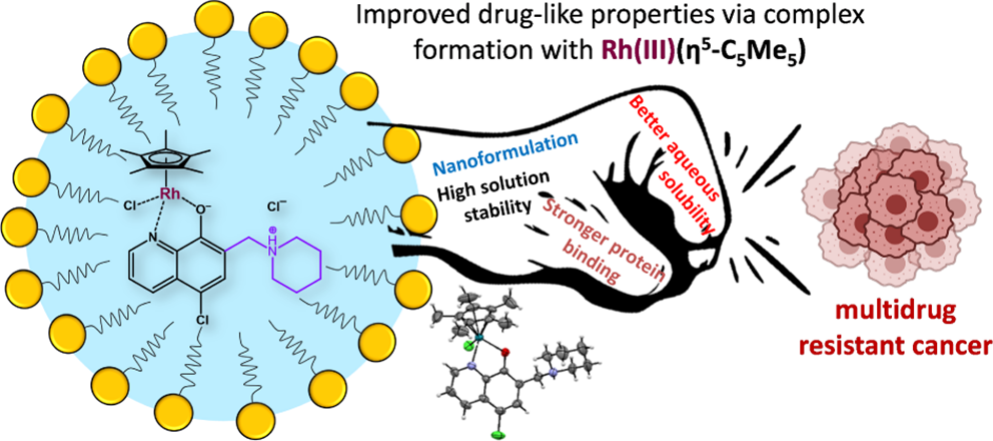

Drug resistance is a major obstacle in cancer treatment. Herein, four novel organometallic complexes, with the general formula [Ru(η^6^-*p*-cymene)(HL)Cl]Cl and [Rh(η^5^-C_5_Me_5_)(HL)Cl]Cl, were developed to target multidrug-resistant (MDR) cancer cells, where HL denotes 8-hydroxyquinoline-derived Mannich bases (HQCl-pyr and HQCl-pip). The aim of the complexation was to obtain compounds with improved drug-like properties. The complexes were comprehensively characterized by various spectroscopic methods in terms of their structure, solution speciation and interaction with human serum albumin. The structure of [Rh(η^5^-C_5_Me_5_)(HQCl-pip)Cl]Cl was analyzed by X-ray crystallography. The complexes were found to be highly stable in solution and in various biological matrices, showing enhanced solubility compared with the ligands and significant binding ability to albumin via coordination. The Rh(η^5^-C_5_Me_5_) complexes exhibited strong cytotoxicity against MDR MES-SA/Dx5 cell lines (IC_50_ = 0.19 and 0.22 μM), demonstrating high MDR-selectivity. Ganglioside-functionalized nanoparticles with the most promising ligand HQCl-pip and its Rh(η^5^-C_5_Me_5_) complex were prepared to enhance the bioavailability. The nanocarriers showed faster drug release at acidic pH than at pH 7.4, and could retain the cytotoxicity and selectivity of the loaded compounds. The encapsulated Rh(η^5^-C_5_Me_5_) complex of HQCl-pip has been identified as an optimal candidate for the pharmacological development of MDR-selective compounds.

## Abstract 2

Two 8-hydroxyquinoline-derived Mannich bases showed potent cytotoxicity and selectivity against multidrug resistant cancer cells. Their half-sandwich Rh(III)(η^5^-C_5_Me_5_) complexes retained the anticancer activity, while the drug-like properties were considerably improved compared to the ligands. One of the ligands and its Rh complex were encapsulated into ganglioside-based nanomicelles to further enhance their bioavailability.

## Introduction

Multidrug resistance (MDR) is considered to be as one of the major obstacles in chemotherapy.^1^ Its development is often associated with the overexpression of ATP-binding cassette transporters, such as P-glycoprotein (ABCB1/P-gp), which leads to reduced drug levels in the intracellular space.^1−3^ Despite clinical failures,^1^ inhibition of the P-gp transporter is still considered a potential strategy to improve therapy.^4^ However, new approaches are also pursued, including research on compounds targeting the collateral sensitivity of P-gp-expressing MDR cancer cells.^5−7^ A large number of compounds capable of selectively killing MDR cells were identified with a large structural diversity, and data mining revealed that their possible mechanism of action is related to interaction with endogenous metal ions.^8^ In particular, we and others have demonstrated that MDR cells exhibit a surprising hypersensitivity to several metal chelating compounds, including isatin-β-thiosemicarbazone,^8,9^ 1,10-phenanthroline,^8,10,11^ and 8-hydroxyquinoline (HQ) derivatives.^5,6,8,10,14−16^ These results highlight the importance of complex formation with essential metal ions such as iron or copper, resulting in the formation of redox-active complexes or the potential depletion of metal ions.^6,16^ Indeed, it was shown, that iron complexation with 8-hydroxyquinoline-derived Mannich bases results in iron depletion in MDR cells via P-gp-mediated efflux of iron complexes.^6,16^ Moreover, a correlation was also found between the p*K*_a_ values of the OH group of the HQ ligand scaffold and cytotoxicity against MDR cells within a library of 120 analogues.^5^ However, a recent study indicated that the MDR-selective toxicity of an 8-hydroxyquinoline-derived Mannich base (MX106-4C) relies on additional mechanisms linked to the P-gp-dependent inhibition of surviving, ATP deprivation and ROS production.^17^ It is noteworthy that HQ derivatives are also recognized for their beneficial medicinal properties,^18−22^ often attributed to their strong metal ion chelating ability.^5,6,8,10,14−16,18−20,22−24^

We have shown that the MDR-selective toxicity of HQs can be effectively improved by incorporating a CH_2_–N subunit at the position 7 on the HQ scaffold, yielding the aforementioned 8-hydroxyquinoline-derived Mannich bases.^5,6,10,25^ These compounds often contain annular tertiary amines at position 7 as well as a halogen (mainly chlorine) substituent at position 5. The compounds have demonstrated strong cytotoxicity against human uterine sarcoma cell lines and exhibited MDR-selective toxicity, with selectivity ratios (SRs) exceeding 10 in some cases.^5,6,25^ However, these compounds are often associated with limited bioavailability and *in vivo* applicability due to their poor aqueous solubility.^12,13^ To increase the hydrophilic character, we introduced polar groups into the HQ backbone, by incorporating proline and homoproline subunits. This modification led to the formation of zwitterions, effectively increasing aqueous solubility.^14,26,27^ While these amino acid conjugates were found to be MDR-selective; their toxicity was impaired due to relatively low membrane permeability.^14,26,27^

**Chart 1:**
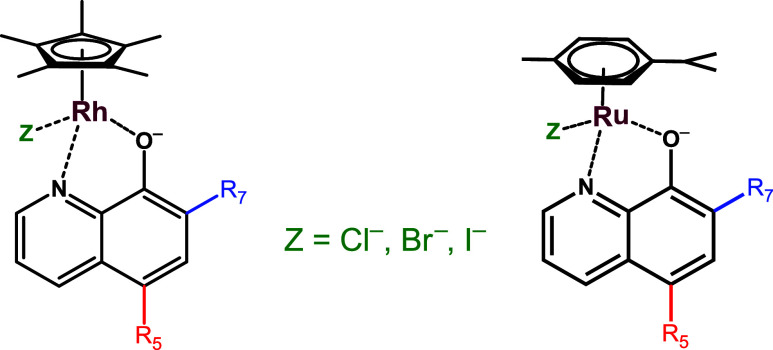
General Chemical Structure of Piano-Stool Half-Sandwich RhCp* and RuCym Complexes Containing HQ Derivatives Substituted at Positions R5 and R7 with a *Z* (Cl^–^, Br^–^, I^–^) Monodentate Co-ligand

**Chart 2:**
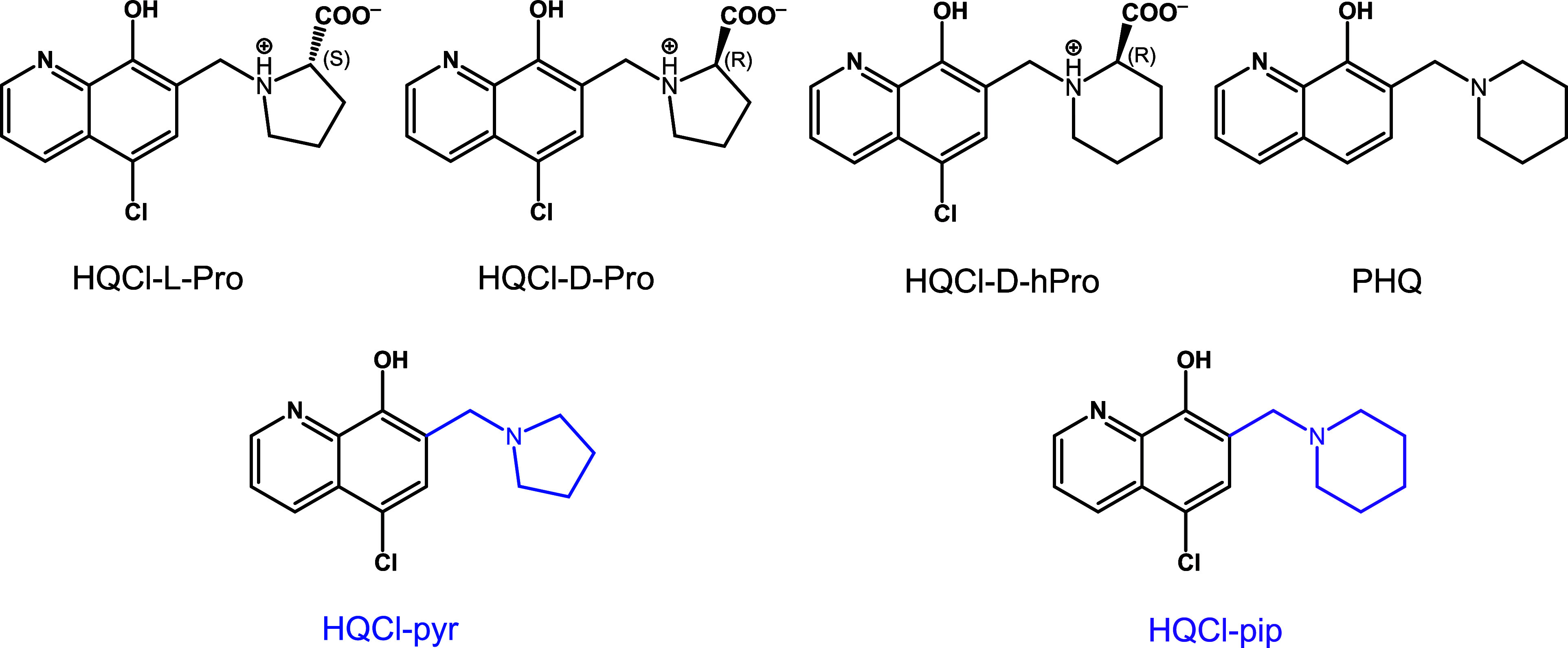
Chemical Structure of Selected 8-Hydroxyquinoline-derived Mannich Bases: HQCl-L-Pro, HQCl-D-Pro, HQCl-D-hPro, and PHQ Studied Previously,^25−27^ and HQCl-pyr, HQCl-pip Studied in This Work Shown in Their HL Form

Complexation with metal ions can be another viable approach to improve drug-like properties, particularly if the resulting complexes exhibit better solubility than the organic ligands. In addition, the high solution stability of the metal complex can facilitate transport of the ligand to the target and the metal center can also modify the mechanism of action. Numerous metal complexes of HQs have been developed and investigated;^26−31^ the complex tris(8-quinolinolato)gallium(III) (KP46) is a well-known representative, demonstrating successful efficacy against renal cancer in clinical trials.^32^ Organometallic complexes such as half-sandwich Ru(η^6^-*p*-cymene) (RuCym) and Rh(η^5^-C_5_Me_5_) (RhCp*) complexes of HQs are also widely investigated due to their remarkable potential in the cancer therapy.^14,25−27,30,31,33−37^ In these piano-stool complexes, the HQ ligand is bound to the metal ion via an (N,O) donor set, and the coordination sphere is saturated by a monodentate coligand (Z), which is usually a halido ligand (Chart 1). The HQ scaffold is often substituted at the positions R5 and R7 due to the *ortho*- and *para*-directing property of the hydroxyl group. We have shown that complexation of HQs containing (homo)proline moieties at position R7 and a chlorine substituent at position R5 (Chart 2) with RhCp* not only resulted in an increased solubility but also improved selectivity toward cancerous cell lines over noncancerous cells.^26^ The complexation with RhCp* complexes of an other Mannich based HQ was found to be advantageous in our previous work,^25^ as the complex of 7-(1-piperidinylmethyl)quinolin-8-ol (PHQ,Chart 2) possessing enhanced hydrophilicity, was highly active on MDR cells.

Additionally, nanoformulation can also significantly increase the bioavailability of drugs by providing protection against enzymatic degradation as well as improving hydrolipophilic properties and cellular uptake. Drug carrier systems, such as inorganic and polymeric nanoparticles, liposomes or nanomicelles are commonly used for targeted drug release.^38−40^ Gangliosides (GMs) are sialic acid-containing glycosphingolipids that are particularly abundant in the plasma membrane of mammalian neurons.^41^ Due to their potential role in the formation of tumor metastases and their overexpression in different types of cancers, they are considered as potential therapeutic targets.^42^ Moreover, these micellar biocompatible molecules have been used as drug carriers also for anticancer compounds,^43−45^ e.g., paclitaxel and doxorubicin were successfully microencapsulated using GM1.^46,47^

Here we report the development of novel RhCp* and RuCym complexes formed with two 8-hydroxyquinoline Mannich bases (5-chloro-7-(pyrrolidin-1-ylmethyl)quinolin-8-ol (HQCl-pyr) and 5-chloro-7-(piperidin-1-ylmethyl)quinolin-8-ol (HQCl-pip)) incorporated a (−CH_2_–N)-pyrrolidine or a (−CH_2_–N)-piperidine subunit at position 7 (Chart 2). A comprehensive investigation of HQCl-pyr and HQCl-pip as well as their organometallic complexes in terms of solution chemical properties and anticancer activity in parental and MDR cancer cells is reported. A ganglioside-based nanomicelle delivery system was developed to improve the bioavailability of the most potent compounds.

## Results and DiscussionS

### Synthesis of the Organometallic Rh(η^5^-C_5_Me_5_) and Ru(η^6^-*p*-cymene) Complexes

The 8-hydroxyquinoline-derived Mannich bases, HQCl-pyr and HQCl-pip (Chart 2), were prepared based on a previous literature method,^48,49^ starting from 5-chloro-8-hydroxyquinoline (HQCl), formaldehyde, and pyrrolidine or piperidine, by implementing different optimization steps as reported in our previous work.^5^ Namely, based on our previous observations, the aminoalkylation was carried out by using aq. formaldehyde solution, and ethanol, as the previously proposed solvent, was replaced by toluene (Scheme S1). ^1^H and ^13^C NMR spectroscopy and electrospray mass spectrometry (ESI-MS) measurements were carried out to confirm the structure and purity of the compounds (see Experimental section and NMR spectra in Figures S1 and 4).

**Chart 3:**
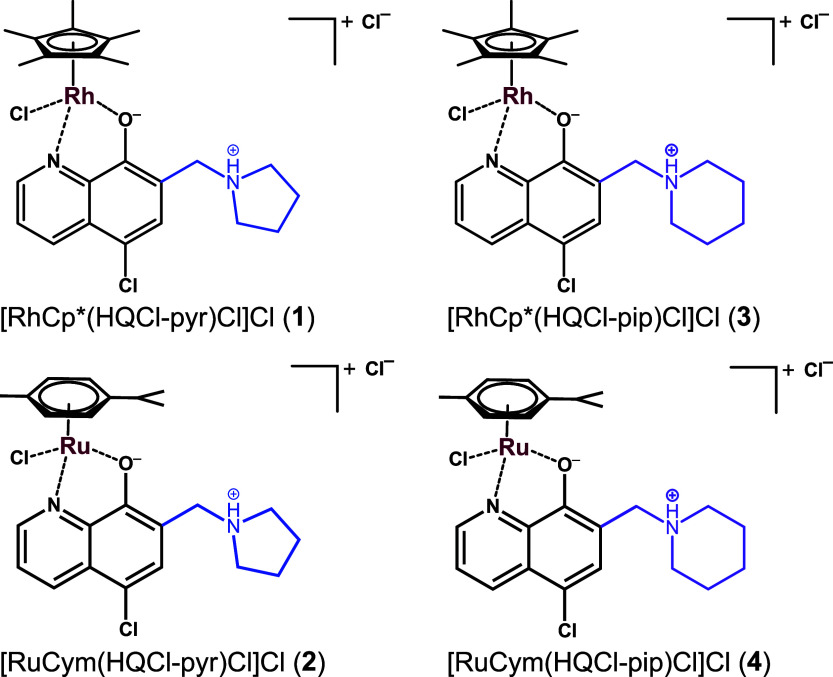
Chemical Structure of the Organometallic RhCp* and RuCym Complexes (**1**–**4**)

The organometallic complexes (**1**–**4**) (Chart 3) were synthesized by mixing the ligands with half-equivalent of the corresponding dimeric precursor [M(arene)Cl_2_]_2_ in CH_2_Cl_2_, where M(arene) is RhCp* in complexes (**1**) and (**3**) and RuCym in (**2**) and (**4**). After 24 h, the solvent was partially evaporated, and precipitation was carried out with diethyl ether. The orange complexes formed were filtered, washed with diethyl ether and *n*-hexane, and then dried at 45 °C for 24 h. ^1^H, ^13^C NMR spectroscopy, and ESI-MS techniques were used to characterize the complexes, yielding the [M (arene)(HL)Cl]Cl formula shown in Chart 3 (see Experimental section and Figures S5–S12), in which the neutral zwitterionic ligand coordinates via the (N,O) donor set and the binding of a chlorido coligand completes the coordination sphere. During the syntheses, the complex formation was accompanied by the simultaneous release of one equivalent of proton, which was bound by the amino group present in the ligand. The additional charge is neutralized by chloride counterion (Chart 3). Capillary zone electrophoresis was also applied to check the purity of the complexes (as an example for complex (**3**) see Figure S13). The lack of unbound ligands and metal precursors in the solid samples was also confirmed by this method. The complex [Rh(III)(η^5^-C_5_Me_5_)(HQCl-pip)Cl]Cl (**3**) was characterized by X-ray crystallography (see the Structural Studies of [RhCp*(HQCl-pip)Cl]Cl × H_2_O × OC_4_ by X-Ray Crystallography).

### Structural Studies of [RhCp*(HQCl-pip)Cl]Cl × H_2_O × OC_4_ by X-Ray Crystallography

**Figure 1:**
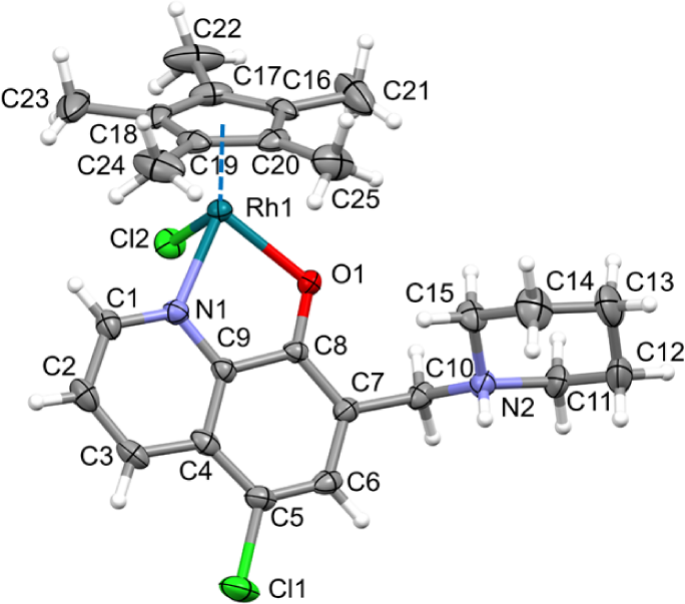
ORTEP representation of crystal [RhCp*(HQCl-pip)Cl]Cl × H_2_O × OC_4_ with atom numbering. Solvent molecules and counterions are omitted for clarity. Displacement parameters are drawn at the 30% probability level.

Single crystals of complex (**3**) as [RhCp*(HQCl-pip)Cl]Cl × H_2_O × OC_4_ were obtained by applying the vapor diffusion method. The previously isolated complex was dissolved in CH_2_Cl_2_ and diethyl ether was allowed to slowly diffuse into the sample. The crystal structure of the complex was determined by single crystal X-ray diffraction (Table S1). It crystallized in the orthorhombic crystal system in the *P*2_1_2_1_2_1_ chiral space group containing a chloride counterion, a solvate water molecule, and a disordered diethyl ether molecule in the asymmetrical unit. (The hydrogen atoms on the carbon atoms of diethyl ether could not be determined). The ORTEP representation of the compound is depicted in Figure 1 and the unit cell containing four complexes is shown in Figure S14.

The Rh(III) ion is coordinated by the Cp* ring, the (N,O^–^) chelate of the HQCl-pip ligand, and a chlorido coligand in a tetrahedral “piano-stool” geometry. Selected bond distances and angles of the coordination sphere are collected in Table S2. The +1 charge of the complex is neutralized by a chloride counterion. Figure S15 shows the linear arrangements of the disordered diethyl ether molecules and the main intermolecular hydrogen bond connections of the solvate molecules and chloride ions with the complex molecules in the crystallographic *a* direction. Details of the main secondary interactions are collected in Table S3. The packing arrangements viewed from the “ab” plane shows the main π···π and C–H···π interactions between neighboring complexes (Figure S16). Figure S17 shows the packing arrangement viewed in the three crystallographic directions. The disordered diethyl ether molecules are placed in channels along the *a* crystallographic axis (Figure S18). The volume of the channel is 204 Å^3^ which is 7.4% of the unit cell volume. It is noteworthy, that in this complex, no intramolecular hydrogen bond could be observed between the O1 and N2 atoms, which was reported for the similar complex [RhCp*(HQCl-D-hPro)Cl]Cl × H_2_O × CH_3_OH^26^ where HQCl-D-hPro is (*R*)-5-chloro-7-((homoproline-1-yl)methyl)8-hydroxyquinoline.

### Solution Chemical Properties of the Ligands and Their Complexes

#### Proton Dissociation Processes and Lipophilicity of HQCl-Pyr and HQCl-Pip

**Figure 2:**
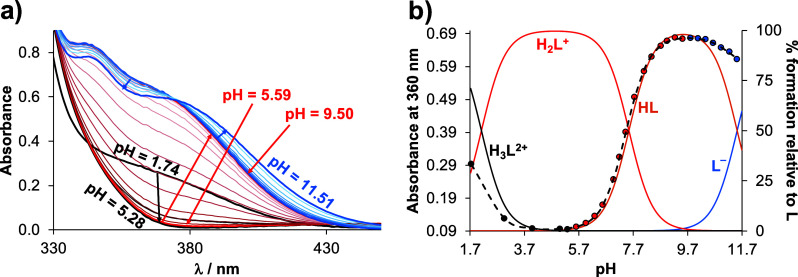
(a) UV–vis spectra of HQCl-pip at increasing pH values and (b) absorbance values at 360 nm (●black circle) plotted against pH with the fitted (dashed) line as well as with the concentration distribution curves (solid lines). The titrations were conducted in the presence of 10 equiv. EDTA. {*c*_HQCl-pip_= 81 μM; *I* = 0.20 M KNO_3_;  = 2 cm; *T* = 25.0 °C}.

Physico-chemical properties such as aqueous solubility, lipophilicity, and the actual protonation state at a given pH are known to influence the pharmacokinetic behavior of a drug. Therefore, the proton dissociation processes of HQCl-pyr and HQCl-pip (Chart 2) were studied by UV–visible (UV–vis) and ^1^H NMR spectroscopic methods in order to determine their p*K*_a_ values. Representative spectra are shown for HQCl-pip in Figure 2, and for S19 and for HQCl-pyr in Figure S20. Ligand HQCl-pip has already been studied in our previous work,^5^ however, here the p*K*_a_ values were redetermined in the presence of the chelating agent ethylenediaminetetraacetic acid (EDTA) to trap the minor metal ion impurity that might influence the equilibrium constants. The p*K*_a_ values calculated on the basis of the pH-dependent spectral changes are shown in Table 1.

**Table 1:** p*K*_a_ Values of HQCl-pyr and HQCl-pip Determined by UV-vis and ^1^H NMR Spectroscopy, in Addition to the Values Predicted {*I* = 0.20 M KNO_3_; *T* = 25.0 °C}

Method	Compound	p*K*_a_ (N_q_H^+^)	p*K*_a_ (OH)	p*K*_a_ (N_pyr/pip_H^+^)
UV–vis	HQCl-pyr	<2	7.53 ± 0.05	11.36 ± 0.10
	HQCl-pip	2.09 ± 0.05	7.42 ± 0.03^a^	11.33 ± 0.12
^1^H NMR	HQCl-pyr	n.d.^b^	7.65 ± 0.02	n.d.^b^
	HQCl-pip	n.d.^b^	7.43 ± 0.02	n.d.^b^
prediction^c^	HQCl-pyr	2.44	7.62	9.26
	HQCl-pip	2.44	7.61	9.14

a p*K*_a_ (OH) = 5.80^5^, *I* = 0.10 M KCl, obtained without the addition of EDTA.

b Based on the changes in the ^1^H NMR spectra recorded between pH 2.3–11.9, only p*K*_a_ (OH) could be determined with adequate accuracy.

c Values predicted by Marvin (Chemaxon) software.^50^

The protonated forms of these HQ derived Mannich bases can be characterized by three proton dissociation processes (Scheme S2), which belong to the deprotonation of the quinolinium–NH^+^ (N_q_H^+^), the phenolic–OH and the pyrrolidinium–NH^+^ (N_pyr_H^+^) (HQCl-pyr) or piperidinium-NH^+^ (N_pip_H^+^) (HQCl-pip) moiety. It is noteworthy that the p*K*_a_ (N_pyr/pip_H^+^) values determined are considerably higher than predicted (Table 1) most likely due to a hydrogen bond formed between the phenolate-O^–^ and the protonated N_pyr/pip_H^+^, as reported for the similar type of HQs based on X-ray diffraction analysis.^15^ Based on the p*K*_a_ values, the fraction of H_2_L^+^ is 51% and 58%, and that of HL is 49% and 42% for HQCl-pyr and HQCl-pip, respectively at pH 7.4. The H_2_L^+^ species consists of protonated OH and N_pyr/pip_H^+^ moieties, while HL is a zwitterion formed by deprotonation of the OH group. It is noteworthy that the value of p*K*_a_ (OH) was different (somewhat higher) in the presence of the EDTA, thus its use is suggested for the adequate determination of the constant, especially when low concentrations are used. Based on the established correlation between p*K*_a_ (OH) values and MDR-selectivity, the relatively lower p*K*_a_ (OH) values of both HQCl-pyr and HQCl-pip suggest high selective toxicity.^5^

The distribution coefficients of HQCl-pyr and HQCl-pip were determined by *n*-octanol/H_2_O partitioning at pH 7.4 (log *D*_7.4_). According to the values determined (log *D*_7.4_ = +1.83 ± 0.02 and +2.54 ± 0.01 for HQCl-pyr and HQCl-pip, respectively), the additional methylene group in HQCl-pip significantly increased lipophilicity. This compound is also more lipophilic than its derivative, PHQ (Chart 2, log *D*_7.4_ = +0.93^25^) due to the presence of the chlorine substituent. HQCl-pip was found to be less water-soluble than HQCl-pyr at pH 7.4, as shown by the thermodynamic aqueous solubility data (*S*_7.4_ = 516 ± 2 and 254 ± 3 μM for HQCl-pyr and HQCl-pip, respectively).

#### Solution Speciation of the Organometallic RhCp* and RuCym Complexes

The stability of the organometallic complexes (**1**–**4**) was first investigated in modified phosphate-buffered saline buffer (PBS) at pH 7.4 by UV–vis spectrophotometry. The complexes showed no significant spectral changes over a period of 48 h (Figure S21); the bidentate ligand remains coordinated in the complexes, however the fast exchange of the chlorido coligand to H_2_O might takes place immediately after dissolution (*vide infra*). The complex formation kinetics was also investigated by UV–vis spectrophotometry at pH ∼5. The spectral changes were followed over time after mixing the organometallic triaqua complex [M(arene)(H_2_O)_3_]^2+^ (100 μM) and the corresponding ligand (100 μM) in a tandem cuvette (not shown). As expected, complex formation in the case of RhCp* reached equilibrium much faster (∼2 min) as compared to RuCym complexes (∼2 h). Therefore, in all cases where the complexes were prepared *in situ* in aqueous solution, we ensured a sufficient waiting time to allow equilibrium to be reached before taking further measurements.

The UV–vis spectra of the complexes prepared *in situ* were then followed at different pH values in the absence of chloride ions. No spectral changes were observed between pH = 2–6.3 (RuCym) or 7.5 (RhCp*), indicating high solution stability in both cases; however, the changes were significant in the basic pH range (Figure S22). In the case of RuCym complexes, two overlapping processes associated with the deprotonation of the coordinated water molecule and the noncoordinated N_pyr/pip_H^+^ moiety were observed. For the RhCp* complexes, these deprotonation processes significantly overlapped, making it impossible to accurately calculate the proton dissociation constants (p*K*_a_). The p*K*_a_ values determined for these processes are shown in Table 2 and Figure S23 together with the values obtained for complexes formed with the amino acid hybrids HQCl-D-Pro and HQCl-D-hPro (Chart 2) for comparison.^26^

**Table 2:** p*K*_a_ Values of the *In Situ* Prepared Complexes (in the Absence of Chloride Ions) and Conditional H_2_O/Cl^–^ Exchange Constants (log*K*′(H_2_O/Cl^–^) of the Complexes {*I* = 0.20 M KNO_3_; *T* = 25.0 °C}

Method	System (1:1)	p*K*_a_ (H_2_O)	p*K*_a_ (N_pyr/pip_H^+^)	log*K*’ (H_2_O/Cl^–^)
UV–vis	RhCp*–HQCl-pyr	n.d.^a^	n.d.^a^	2.23 ± 0.02
	RhCp*–HQCl-pip	n.d.^a^	n.d.^a^	2.21 ± 0.03
	RuCym – HQCl-pyr	8.42 ± 0.03	10.32 ± 0.03	1.60 ± 0.07
	RuCym–HQCl-pip	8.39 ± 0.10	10.06 ± 0.05	1.78 ± 0.02
^1^H NMR^b^	RuCym–HQCl-pip	8.50 ± 0.02	10.19 ± 0.02	–
pH-potentiometry	RhCp*–HQCl-pyr	9.35 ± 0.03	10.47 ± 0.03	–
	RhCp*–HQCl-pip	9.39 ± 0.07	10.41 ± 0.10	–
	RuCym–HQCl-pyr	8.47 ± 0.04	10.40 ± 0.03	–
	RuCym–HQCl-pip	8.43 ± 0.08	10.07 ± 0.07	–

a Not determined due to overlapping processes.

b ^1^H NMR titration was also carried out for the RhCp*-HQCl-pip (1:1) system, but p*K*_a_ values could not be reliably calculated due to the strong overlapping processes.

**Figure 3:**
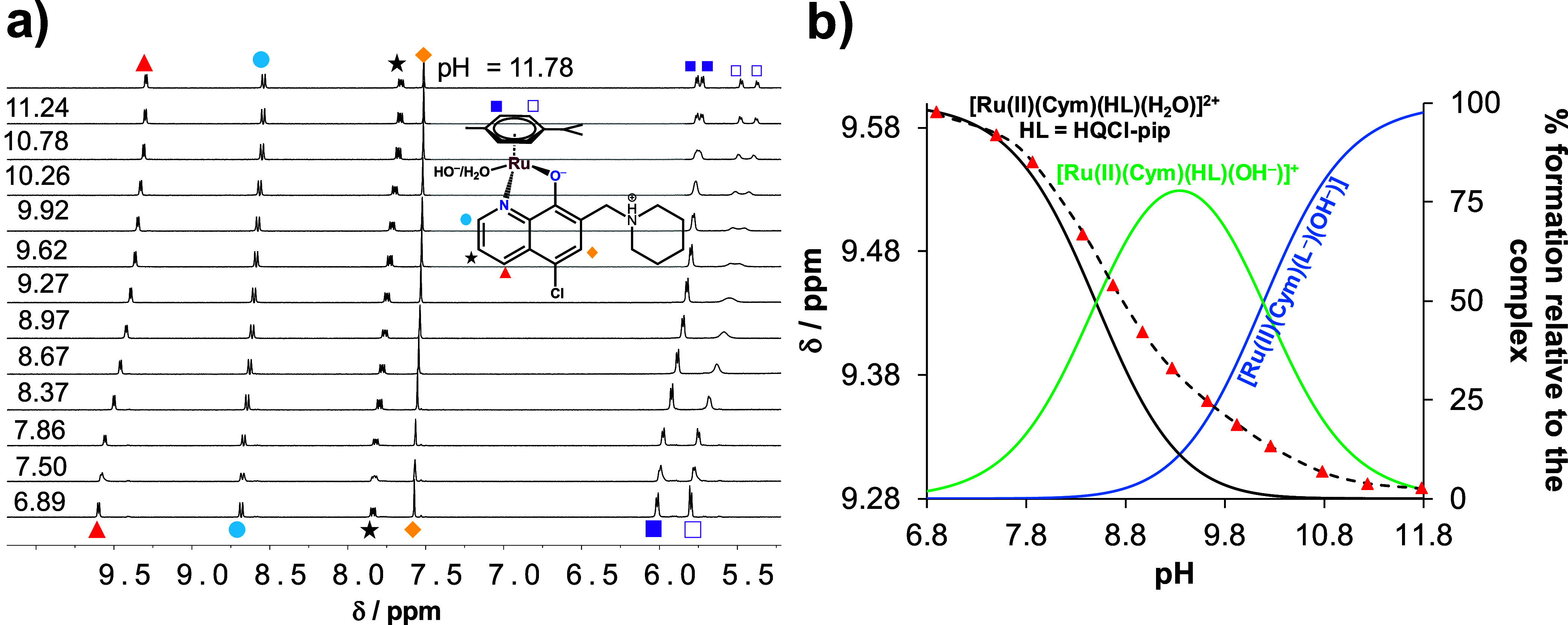
(a) ^1^H NMR spectra at the downfield region of the [RuCym(H_2_O)_3_]^2+^–HQCl-pip (1:1) system at different pH values (6.93 → 11.82). (b) Chemical shifts value of C^4^H proton (red triangle) along with the fitted (dashed) line as a function of pH as well as with the concentration distribution curves (solid lines). {*c*_RuCym_= *c*_ligand_= 1.55 mM, *I* = 0.20 M KNO_3_, 10% (v/v) D_2_O/H_2_O, *T* = 25.0 °C}.

Due to the good solubility of the complexes in water, ^1^H NMR spectroscopic (only for the RuCym–HQCl-pip and RhCp*–HQCl-pip systems, Figures 3 and S24, respectively) and pH-potentiometric titrations were also carried out and, where possible, p*K*_a_ values were calculated. The results, shown in Table 2, indicate good agreement between the data obtained by the different methods. It should be noted that these processes show overlap in all cases, and therefore, the assignment of the p*K*_a_ values to the given moieties cannot be made reliably. However, by comparing the spectral changes with those observed for HQ and other simple derivatives,^25^ it can be concluded that the lower p*K*_a_ likely belongs to the deprotonation of the aqua coligand.

The RhCp* complexes have significantly higher p*K*_a_ (H_2_O) values than the corresponding RuCym complexes, consistent with findings of our former publications.^14,26,27^ The relatively high p*K*_a_ (H_2_O) values resulted in only a minor fraction of the mixed hydroxido species at pH = 7.4 even in the case of the RuCym complexes. These p*K*_a_ (H_2_O) values are lower than those of the complexes formed with the corresponding amino acid hybrids (HQCl-D-Pro, HQCl-D-hPro, Figure S23). Thus, the predominant complex at pH 7.4 is [M(arene)(HL)(H_2_O)]^2+^ in all cases, in which the bidentate ligand coordinates via the (N,O^–^) donor set and the pyridinium or pyrrolidinium nitrogens are protonated just like in the isolated complexes (Chart 3). Based on the unchanged spectra (*vide supra*), these types of complexes show no dissociation at pH = 2, indicating their high stability and hindering the direct determination of the formation constants. Therefore, a ligand displacement experiment was performed using UV–vis spectrophotometry with 2,2′-bipyridine (bpy) as a competitor to determine conditional formation constants at pH = 7.4 (Figure S25). The calculated equilibrium constants are log*K*’ [M(arene)L]_7.4_ = 12.06 ± 0.02 and 11.89 ± 0.03 for RhCp* complexes of HQCl-pyr and HQCl-pip, respectively, which are lower compared to those of the HQCl-D-(h)Pro complexes Figure S23.^26^ Nevertheless, these values indicate a high stability of the complexes in solution, and <1% of dissociation is estimated at 1 μM concentration at pH = 7.4. The ligand displacement measurement could not be applied to the RuCym complexes due to the release of the *p*-cymene ring followed by the formation of the mixed ligand complex with bpy and oxidation of the metal center (Figure S26), similarly to analogue complexes.^14,26,27^

When the isolated complexes (**1**–**4**) are dissolved in water, their chlorido coligand can be replaced by water, or *vice versa* in the aqua complexes [M(arene)(HL)(H_2_O)]^2+^, the H_2_O → Cl^–^ exchange process can take place in the presence of chloride ions. The chloride ion affinity of the complexes was also investigated by UV–vis spectrophotometry. RhCp* and RuCym complexes were titrated with chloride ions at pH 7.4 and 6.0, respectively. For this experiment, pH values were chosen at which the formation of the mixed hydroxido species is negligible. A representative spectrum series for complex (**4**) is shown in Figure S27, and the calculated conditional exchange constants (log*K*’ (H_2_O/Cl^–^)) are given in Table 2 and in Figure S23. The data indicate a higher chloride ion affinity of the half-sandwich RhCp* complexes over RuCym complexes, as found previously.^14,25−27,51^ The complexes of the amino acid hybrids HQCl-D-Pro and HQCl-D-hPro have lower log*K*’ (H_2_O/Cl^–^) constants most likely due to the repulsive effect of the nearby COO^–^ group.^26^ The inverse correlation between the p*K*_a_ (H_2_O) and log*K*’ (H_2_O/Cl^–^) values were seen for a set of half-sandwich RhCp* and RuCym complexes bearing different bidentate ligands,^14,25−27,51^ which is also clearly seen in Figure S23 for the complexes studied here.

**Figure 4:**
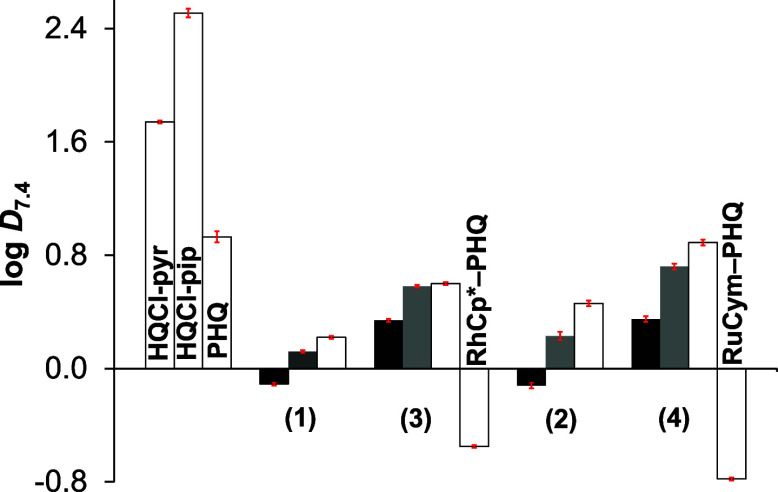
Distribution coefficients (log*D*_7.4_) of the ligands and their organometallic complexes determined by *n-*octanol/H_2_O partitioning at pH = 7.4 at three different chloride ion concentrations: 4 (black bars), 24 (gray bars), and 100 mM (white bars) relevant to nucleus, cytosol, and blood plasma, respectively. Previously published data^25^ for PHQ and its RhCp* and RuCym complexes are also shown at 100 mM chloride ion concentration, for comparison. The values are given in Table S4. {*c*_complex_ = 200 μM; *T* = 25.0 °C}.

The exchange of the coligand (H_2_O/Cl^–^) changes the overall charge of the complex, thus affecting lipophilicity. To characterize the lipophilicity of the complexes, distribution coefficients (log *D*) were determined at pH = 7.4 at three different chloride ion concentrations with respect to different biofluids (4, 24, and 100 mM for nucleus, cytosol and blood plasma, respectively) using the traditional *n*-octanol/water partitioning method (Table S4 and Figure 4).

When the chlorido ligand is coordinated in complexes (**1**–**4**) at pH 7.4, the charge of the complex [M(arene)(HL)(Cl)]^+^ is +1, and becomes +2 when the Cl^–^ → H_2_O exchange occurs. As expected, when the chloride ion concentration of the medium was increased, the complexes became more lipophilic due to their decreased charge. The complexes formed with HQCl-pip (**3**, **4**) are more lipophilic compared to HQCl-pyr complexes (**1**, **2**), due to the presence of the additional methylene group. Compounds (**3**, **4**) are more lipophilic than their analogous PHQ complexes without the chlorine substituent at position 5 (Figure 4). Comparing the log*D*_7.4_ values of the compounds (**1**–**4**) with those of the ligands HQCl-pyr/pip (Figure 4), it is clear that the half-sandwich complexes are significantly more hydrophilic, which enhances their applicability *in vivo*.

**Figure 5:**
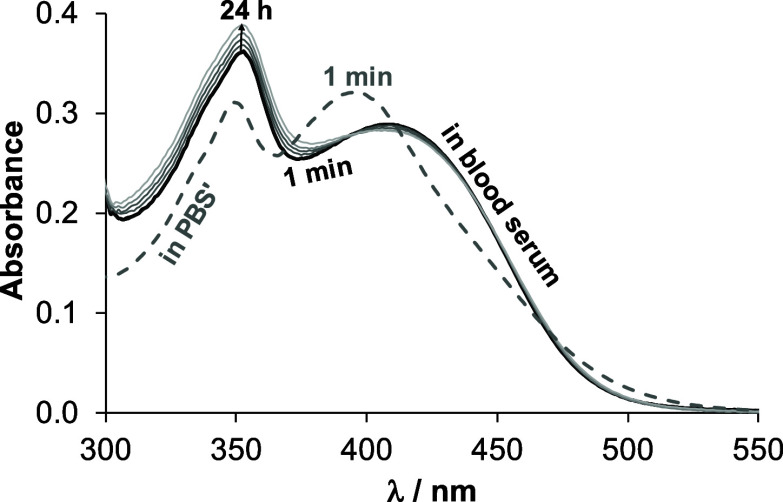
UV–vis spectra of complex (**3**) ([RhCp*(HQCl-pip)Cl]Cl) in PBS’ recorded after 1 min after dissolution (gray dashed line) and in real blood serum (black and gray solid lines). {*c*_complex_= 100 μM; in PBS’ or in blood serum 4-fold diluted with PBS’ (pH = 7.4);  = 1 cm; *T* = 25.0 °C}.

The stability of the complexes (**1**–**4**) was also studied in blood serum and in cell culture Eagle’s Minimum Essential Medium (EMEM) by UV–vis and ^1^H NMR spectroscopic methods. Comparing the UV–vis spectrum of the complexes in PBS’ and in blood serum, a relatively rapid (∼1 min) process followed by a slower one could be observed for all complexes (see Figure 5 for complex (**3**)). It is likely that the complexes are able to interact with blood serum component(s), leading to changes in the charge transfer bands. However, based on the recorded spectra, ligand release is unlikely. Instead, binding via exchange of the chlorido coligand with a donor atom of the serum ligand is suggested. The UV–vis spectra recorded for the complexes in EMEM also indicated changes, however, the ^1^H NMR spectra clearly suggest that the complexes retain the original bidentate ligand in the medium due to their high stability (only the coligand is exchanged). It is likely that only binding of a medium-component occurs (see Figure S28 for complex (**3)**).

#### Interaction of the Organometallic Complexes with Human Serum Albumin

As the complexes showed changes when dissolved in blood serum (*vide supra*), their interaction was studied with the most abundant blood protein, human serum albumin (HSA). This protein plays a key role in transporting many different types of endogenous and exogenous compounds. Binding of a drug molecule to HSA can significantly influence its pharmacokinetic properties and serve as a targeting vehicle due to the enhanced permeability and retention effect.^52^

**Figure 6:**
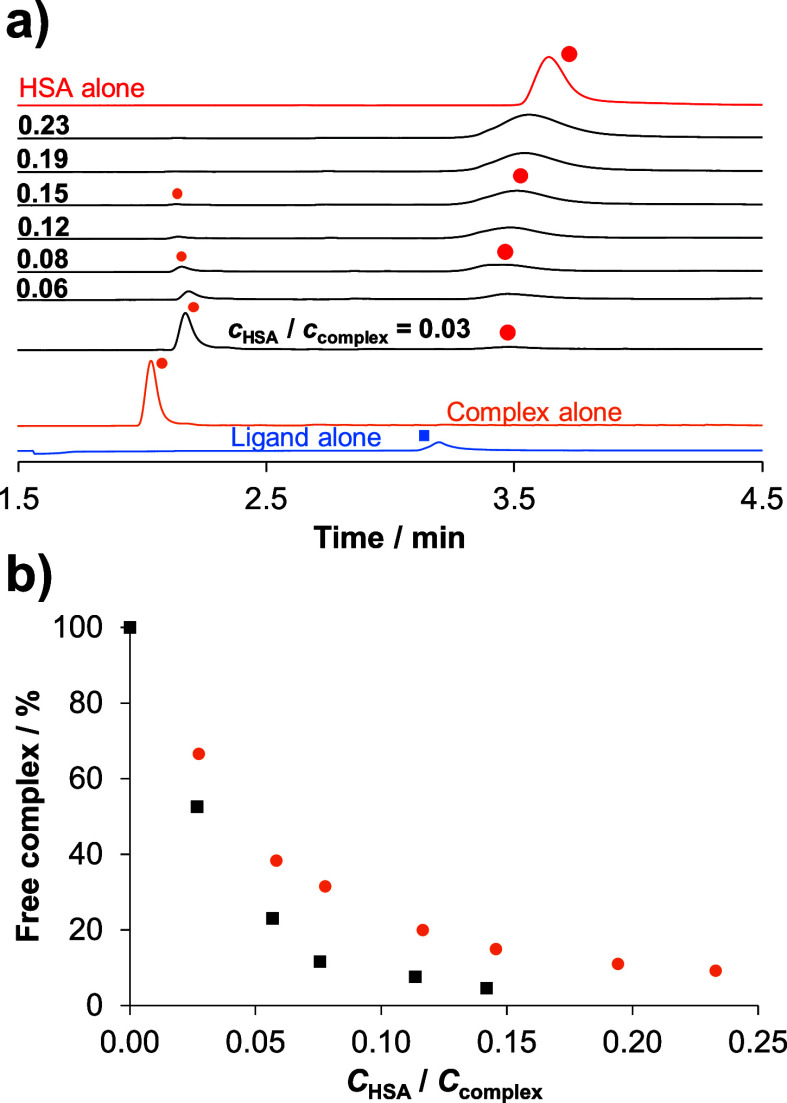
(a) Electropherograms of free HQCl-pip, complex (**4**), HSA and the complex in the presence of HSA at various complex-to-HSA ratios after a 24 h incubation time. Symbols indicate the free ligand HQCl-pip (blue square), free complex (**4**) (orange dot), and HSA and HSA-complex adduct (red dot). Peaks belonging to the free complex were assigned based on their UV–vis spectra measured at the peak maxima. (b) Fraction of the free (unbound) complex for (**2**) (black square) and for (**4**) (orange dot) under the same condition. {*c*_HSA_ = 20 μM; *c*_complex_ = 86–729 μM; pH = 7.4 (PBS’ buffer); *λ*= 200 nm; *T* = 25.0 °C; waiting time: 24 h}.

The interaction of the organometallic complexes (**1**–**4**) with HSA was investigated by the combined use of capillary zone electrophoresis (CZE), spectrofluorimetry, and UV–vis spectrophotometry. Since the ligand-exchange processes for the RuCym complexes were found to be very slow, a waiting period of 24 h was applied for all experiments in this section. The use of CZE is justified because the electrophoretic mobilities of the free and the protein-bound complex (that appears with the signal of the protein) differ, as evidenced by Figure 6a showing representative electropherograms recorded for the complex (**4**). Based on the peak integrals, a small fraction of the complex remains unbound at ∼0.2 equiv of HSA, similarly to what was observed for e.g., complex (**2**), indicated in Figure 6b. This bound complex/protein ratio also suggests that the number of the bound complexes can be even higher than 4. As no free ligand was detected, the binding of the complex with the bound bidentate ligand is suggested. It is likely that the monodentate coordination of a side chain donor atom of HSA occurs at the coligand binding site.

The changes in the UV–vis spectra of complex (**4**) in the presence of varying equiv. HSA (Figure S29) demonstrate that the charge transfer bands are significantly shifted due to the interaction with the protein. This observation also suggests that no ligand is released; the complex is bound to the protein by coordination rather than intermolecular bonds. The absorption spectra showed changes only up to ∼0.2–0.25 equiv HSA for complexes (**3**) and (**4**), indicating that the maximum number of the bound complexes is consistent with the CZE results.

Surface-exposed His side chain residues are thought to be the primary binding sites for these types of organometallic complexes.^53−57^ Therefore, 1-methylimidazole (MIM) and terminally protected tripeptides such as Ac-Ala-His-Ala-NH_2_ (AHA), Ac-Phe-His-Ala-NH_2_ (FHA) oligopeptides were used as models for HSA binding, as employed in our former studies.^14,26,53^ Representative UV–vis spectra for complex (**4**) in the presence of different equiv. MIM is shown in Figure S30, revealing spectral changes that are rather similar to those obtained for HSA. These results confirm that coordinative binding in the HSA-metal complex adduct is due to the binding of a His imidazole nitrogen of the protein. From the spectral changes, log*K*’ conditional formation constants for the adducts were calculated (Table 3), showing that the RuCym complexes exhibit a higher affinity for MIM compared to RhCp* complexes. A similar trend was observed for the binding of FHA and AHA.

**Table 3:** Formation Constants of the Adducts Formed with the HSA Binding Models MIM, FHA and AHA determined by UV-vis Spectrophotometry and Quenching (Trp214) and DG Displacement Equilibrium Constants (log*K*’) of the Organometallic Complexes (**1**) – (**4**) obtained from Spectrofluorometry Measurements upon Binding to HSA. {*c*_HSA_ = *c*_DG_ = 1 μM, *c*_complex_ = 0–50 μM, λ_EX_ = 295 nm (Trp-214) or *λ*_EX_ = 335 nm (DG); pH = 7.4, (PBS’); *T* = 25.0 °C; Waiting time: 24 h}

Complex	log*K*’_(MIM)_	log*K*’_(FHA)_	log*K*’_(AHA)_	log*K*’_(quenching)_^a^	log*K*’_(DG)_^b^
(**1**)	4.50 ± 0.07	4.52 ± 0.02	4.30 ± 0.01	5.1 ± 0.1	5.0 ± 0.1
(**3**)	4.48 ± 0.12	4.52 ± 0.01	4.13 ± 0.01	5.1 ± 0.1	5.0 ± 0.1
(**2**)	5.85 ± 0.03	5.31 ± 0.01	5.39 ± 0.02	5.3 ± 0.2	5.2 ± 0.1
(**4**)	5.86 ± 0.07	5.38 ± 0.04	5.37 ± 0.02	5.4 ± 0.2	5.3 ± 0.1

a log*K*’_(quenching)_ = 4.6 ± 0.1 for HQCl-pyr, and 5.1 ± 0.1 HQCl-pip.

b log*K*’_(DG)_ = 4.9 ± 0.1 for HQCl-pyr, and 5.4 ± 0.1 HQCl-pip.

Next, spectrofluorometric measurements were also performed to gain insight into the binding interactions that may occur in the hydrophobic binding sites of HSA. Tryptophan (Trp214) quenching experiments were conducted for the complexes (**1**–**4**) (for complex (**3**) see Figure S31) and their ligands). The quenching constants (Table 3) were found to be fairly similar to the exception of HQCl-pyr, which has a lower value. Notably, the complexes showed complete quenching (with the intensity decreasing to zero upon the addition of the complex), whereas the ligands induced only partial quenching (up to a maximum of 40%). As Trp214 is more sensitive to binding to site I than to binding at other sites, these results suggest that the complexes are able to interact at this particular site, meanwhile other sites may be the main binding sites for the ligands. To monitor the binding at site II, site marker displacement experiments were performed using dansylglycine (DG). The displacement constants (Table 3) for the complexes were similar to each other and to the quenching constants, indicating that they are able to bind close to both sites. In all, the organometallic complexes (**1**–**4**) are able to bind to HSA via a coordinative binding mode at multiple binding sites.

#### Ganglioside-based Nanomicelles: Preparation and Characterization

Nanomicelles are considered as effective pharmaceutical carriers for solubilizing hydrophobic drugs, and their outer hydrophilic corona protects the construct from recognition and removal by the reticuloendothelial system, resulting in extended circulation times.^58^ Additionally, nanomicelles disintegrate very slowly due to their inertness, which helps them maintain their integrity and drug content until they reach their target site, thereby increasing the drug’s bioavailability.^59^ Gangliosides are amphiphilic materials, so they can form nanomicelles and entrap hydrophobic molecules or compounds containing a hydrophobic part. It should be also noted that ganglioside nanomicelles typically have very low critical micelle concentration, ranging from 10^–10^ to 10^–8^ M, which enables them to maintain their micellar structure after multiple dilutions.

**Figure 7:**
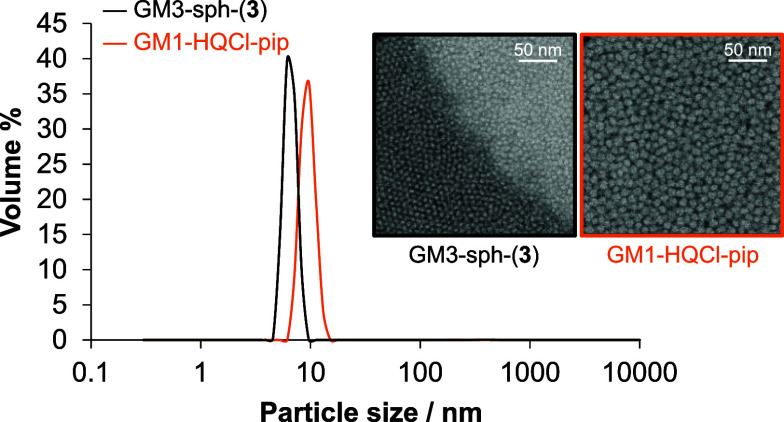
Particle size distribution by volume of GM1-HQCl-pip (orange line) and GM3-sph-(**3**) (black line) nanomicelles. Inserted figures show their S/TEM images.

GM1 and GM3-sphingosine (GM3-sph) gangliosides were selected for the encapsulation of the most promising compounds, HQCl-pip, and its RhCp* complex, (**3**), respectively. GM1 nanomicelles with HQCl-pip and GM3-sph nanomicelles, including the complex (**3**), were formed spontaneously in the salt solutions. The encapsulation efficiency (EE%) of (**3**) by GM3-sph was extremely high 95.5 ± 2.6%, while EE% for HQCl-pip into GM1 was 49.1 ± 2.0%. The size distributions by volume for the prepared micelles are listed in Figure 7. GM1- HQCl-pip nanomicelles had a mean diameter of 11.9 nm, while this value was 8.3 nm for GM3-sph-(**3**) micelles.

The size of GM1-HQCl-pip nanomicelles is comparable to the average size of 10–12 nm found by others for other anticancer drugs entrapped by GM1 nanomicelles, including paclitaxel^45^ or doxorubicin.^60^ In the literature, we found information only on the size of GM3-ceramide, which has an average diameter of ∼25 nm.^61^ GM3-ceramide contains an amide bond linked to a stearic acid, whereas GM3-sph contains free amino groups. In our measurements, GM3-sph with the organometallic complex was substantially smaller compared with GM3-ceramide and ligand-loaded GM1. Scanning/transmission electron microscopy (S/TEM) images (Figure 7) confirmed that the GM3-sph formed substantially smaller nanomicelles compared to GM1. The GM1-HQCl-pip nanomicelles exhibited an almost spherical morphology, while GM3-sph-(**3**) nanomicelles were characterized by a rough surface.

**Figure 8:**
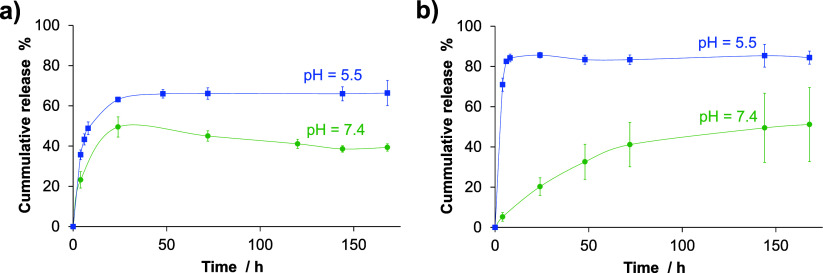
Release kinetics of a) HQCl-pip and b) complex (**3**) from GM1 and GM3-sph nanomicelles, respectively at pH 7.4 (PBS) and pH 5.5 (CH_3_COOH/CH_3_COONa buffer). {*T* = 37.0 °C}.

Both the GM1-HQCl-pip and GM3-sph-(**3**) nanomicelles showed strong electrostatic stabilization, as indicated by their Zeta potential values of −39.2 ± 0.6 and −44.9 ± 1.3 mV, respectively. Drug release experiments from nanomicelles were carried out by the dialysis method using UV–vis spectrophotometry for concentration determination. The release test was performed under normal physiological conditions (pH 7.4, PBS) and in an acidic environment (pH 5.5, CH_3_COOH/CH_3_COONa buffer) at 37 °C (Figure 8). A significantly faster release of the encapsulated compounds was observed from both types of nanomicelles at pH 5.5, which is characteristic of the tumor microenvironment, compared to pH 7.4. This is advantageous as it ensures that drug release is minimized in the blood and enhanced at the targeted tumor site. Both compounds reached a plateau after 24 h in acidic medium, while complex (**3**) demonstrated a continuous, sustained release of the complex at pH 7.4. GM1-HQCl-pip was not stable at pH 7.4 after 24 h, as evidenced by the decrease in the release, indicating its degradation.

#### Anticancer and Antibacterial Activity of the Complexes (1–4)

HQCl-pyr and HQCl-pip have been tested previously for their cytotoxicity on the human uterine sarcoma cell line MES-SA and its doxorubicin-selected MDR derivative MES-SA/Dx5, as well as on the epidermoid carcinoma cell line A431 and its retrovirally transduced P-gp overexpressing counterpart.^5^ The IC_50_ and selectivity ratio (SR) values determined on the two cell pairs (Table S5) revealed the MDR-selective toxicity of these Mannich base derivatives, indicating that they can selectively target P-gp-expressing multidrug resistant cancer cells of different origin (data for PHQ (the HQCl-pip analogue without the chlorine substituent at position 5) are shown for comparison).^25^ The cytotoxicity of HQCl-pyr and HQCl-pip was also assessed in this work on the human colon adenocarcinoma cell line Colo205 and its doxorubicin-resistant counterpart Colo320. The IC_50_ values (Table S5) further confirmed the robust MDR selectivity of these compounds. These experiments used conventional monocultures; however, coculture systems composed of two (or more) different cell types can offer several advantages, such as better mimicking of the tumor microenvironment and allowing interactions between cell lines. Therefore, the novel half-sandwich complexes (**1**–**4**) and their corresponding ligands were tested against a coculture of MES-SA and MES-SA/Dx5 cells expressing the fluorescent proteins mCherry or eGFP, respectively.^62^ As indicated by the IC_50_ and SR values in Table 4, the ligands demonstrated greater cytotoxicity against the MES-SA/Dx5 cells. As compared to PHQ, HQCl-pyr and HQCl-pip showed increased selectivity against MDR cells, consistent with our previous observations in monocultures.^5,25^ As expected, in the presence of the specific P-gp-inhibitor tariquidar (TQ), the IC_50_ values remained nearly unchanged in the parental P-gp-negative MES-SA cells. However, they were significantly increased in MES-SA/Dx5 cells, proving that the cytotoxicity is indeed potentiated by this drug-efflux pump.

**Table 4:** Cytotoxicity of the RhCp* (**1**, **3**) and RuCym (**2**, **4**) Complexes and Their Ligands Measured in a Coculture of MES-SA and MES-SA/Dx5 Cell Lines Expressing the Fluorescent Proteins mCherry and eGFP, Respectively^a^

	MES-SA		MES-SA/Dx5		
ligand alone	IC_50_	+SD/–SD	IC_50_	+SD/–SD	SR
HQCl-pyr	2.94	0.43/0.37	0.25	0.10/0.07	11.6
HQCl-pip	1.09	0.15/0.13	0.09	0.01/0.01	11.8
PHQ	3.80	1.35/1.00	0.49	0.21/0.15	7.8
HQCl-pyr + TQ	2.55	0.55/0.45	1.51	0.59/0.43	1.7
HQCl-pip + TQ	0.88	0.05/0.05	0.71	0.02/0.02	1.2
PHQ + TQ	3.91	1.23/0.94	2.18	0.64/0.50	1.8
RhCp* complex					
HQCl-pyr (**1**)	2.97	0.68/0.56	0.19	0.05/0.04	16.0
HQCl-pip (**3**)	2.04	0.53/0.42	0.22	0.04/0.03	9.1
HQCl-pyr (**1**) + TQ	3.11	0.09/0.08	2.47	0.20/0.18	1.3
HQCl-pip (**3**) + TQ	1.69	0.32/0.27	1.26	0.09/0.08	1.3
***RuCym complex***					
HQCl-pyr (**2**)	8.97	1.74/1.46	2.78	0.78/0.61	3.2
HQCl-pip (**4**)	3.46	0.61/0.52	1.92	0.20/0.18	1.8
HQCl-pyr (**2**) + TQ	3.86	0.74/0.62	38.02	0.89/0.87	0.1
HQCl-pip (**4**) + TQ	2.57	0.10/0.09	13.80	1.33/1.21	0.2
doxorubicin	0.03	0.01/0.01	2.62	0.64/0.51	0.01
doxorubicin + TQ	0.02	0.01/0.01	0.02	0.01/0.01	1.3

a Data for PHQ are shown for comparison.^25^ IC_50_ values (μM) and standard deviations (SD) were calculated from the pIC_50_ (= –logIC_50_) values. Selectivity ratio (SR) was calculated as IC_50_ (MES-SA)/IC_50_ (MES-SA/Dx5). TQ and TS denotes the presence of 0.4 μM tariquidar. Doxorubicin was used as a positive control.

As compared to their respective ligands, RhCp* complexes (**1**) and (**3**) displayed similar cytotoxicity and SR values, while the structurally similar RuCym complexes exhibited higher IC_50_ and lower SR values (Table 4). This phenomenon has already been observed for related half-sandwich organometallic complexes, as PHQ and its complexes (Table S5) also showed a similar cytotoxicity and selectivity profile.^14,25,26^ The weaker cytotoxic activity of the RuCym complexes is thought to be due to their susceptibility to arene loss and subsequent oxidation, rendering the complexes more inert.^14,25,26^ Selective cytotoxicity of the RhCp* complexes (**1**) and (**3**) was abolished in the presence TQ, similar to the effect observed with the ligands, while the cytotoxicity of RuCym complexes (**2**) and (**4**) decreased beyond the cytotoxicity values against the parental line (Table 4). The similar behavior of the ligands and their corresponding RhCp* complexes regarding the IC_50_ and SR values with and without TQ suggests a similar mechanism of action. As for this type of Mannich based HQs it was suggested that their iron complexes are effluxed by P-gp, leading to iron deprivation,^6,16^ we assume that these RhCp* complexes can release the bidentate HQ ligand intracellularly in spite of their high thermodynamic stability. However, further biological studies are needed to prove this suggestion.

As compared to HQCl-pyr, the presence of the additional methylene group in HQCl-pip resulted in lower IC_50_ values in the case of both the ligands and the complexes (Table 4) most probably due to the increased lipophilicity (Figure 4). Thus, HQCl-pip and its RhCp* complex (**3**) were selected to form ganglioside-based nanomicelles (*vide supra*). The nanoformulated products GM1-HQCl-pip and GM3-sph-(**3**), along with the respective ligand (HQCl-pip) and complex (**3**) were tested in a triple coculture comprising the parental MES-SA cells, the doxorubicin-selected MDR cell MES-SA/Dx5 and MES-SA/B1 cells engineered to overexpress P-gp, each expressing different fluorescent proteins to allow multiplexing.^62^ The cytotoxicity data (Table 5) indicated that the nanoformulation did not significantly alter IC_50_ values, suggesting that both the ligand and complex remained accessible to cancer cells. Furthermore, the formulations retained their MDR-selective activity, effectively eliminating both MDR cell lines.

**Table 5:** Cytotoxicity of the Ganglioside-based Nanomicelles (GM1-HQCl-pip, GM3-sph-(**3**)) Against a Triple Coculture of MES-SA, MES-SA/B1 and MES-SA/Dx5 Cells, Expressing the Fluorescent Proteins mCherry, mOrange, or eGFP, Respectively^a^

	MES-SA		MES-SA/Dx5		MES-SA/B1		SR	SR
ligand alone	IC_50_	+SD/–SD	IC_50_	+SD/–SD	IC_50_	+SD/–SD	Dx5	B1
HQCl-pip	1.11	0.41/0.30	0.11	0.04/0.03	0.38	0.19/0.13	10.2	2.9
GM1-HQCl-pip	1.60	0.40/0.32	0.13	0.01/0.01	0.61	0.02/0.02	12.2	2.6
RhCp* complex								
HQCl-pip (**3**)	2.11	0.60/0.47	0.19	0.05/0.04	0.74	0.36/0.24	11.1	2.9
GM3-sph-(**3**)	2.17	1.10/0.70	0.17	0.09/0.06	0.89	0.31/0.23	12.9	2.4
doxorubicin	0.03	0.01/0.00	1.07	0.35/0.27	n.d.	–	0.03	–
etoposide	0.26	0.07/0.06	1.7	0.47/0.37	1.17	0.33/0.26	0.15	0.22

a HQCl-pip and complex (3) were tested for comparison. IC_50_ values (μM) and standard deviations (SD) were calculated from pIC_50_ values. Selectivity ratio (SR) was calculated as IC_50_ (MES-SA)/IC_50_ (MES-SA/Dx5) or IC_50_ (MES-SA)/IC_50_ (MES-SA/B1). Cytotoxicity of doxorubicin (used as positive control) against MES-SA/B1 was not possible due to its overlapping fluorescence with mOrange; thus, etoposide, a non-fluorescent P-gp substrate, was also tested.

The antibacterial activity of the ligands and their complexes was studied on the Gram-negative *Escherichia coli* and the Gram-positive *Staphylococcus aureus* strains (Table S6). The ligands and their RhCp* complexes showed a moderate antimicrobial effect (minimum inhibitory concentration (MIC) = 50 or 25 μM in *Escherichia coli* and *Staphylococcus aureus*, respectively) in all bacterial strains. Remarkably, HQCl-pip and its RhCp* complex (**3**) exhibited the most potent activity against the methicillin-resistant *S. aureus* (MRSA) strain (MIC = 25 μM), a notorious human pathogen accountable for numerous difficult-to-treat hospital-acquired infections.^63^ The diminished pharmacological activity of the RuCym complexes (MIC > 100 μM) was also observed in these bacterial strains, most probably due to the aforementioned arene loss and oxidation.

## Conclusions

In this work, we report the synthesis and characterization of four novel organometallic half-sandwich complexes Ru(II)(η^6^-*p*-cymene)(HL)Cl]Cl and [Rh(III)(η^5^-C_5_Me_5_)(HL)Cl]Cl formed with 8-hydroxyquinoline derived Mannich base ligands (HL = HQCl-pyr and HQCl-pip) in addition to the studies of their solution chemical properties and pharmacological activity. Crystal structure of complex (**3**) was determined using single-crystal X-ray diffraction analysis, revealing the typical piano-stool geometry, in which the coordination sphere is saturated by the bidentate ligand with the (N,O) chelating donor atom set as well as the chlorido coligand. The ligands are found in both their neutral and positively charged forms at a pH of 7.4 in roughly equal amounts, contributing to their lipophilic character. Introducing an additional methylene group in HQCl-pip notably increases its lipophilicity compared to HQCl-pyr, which is consistent with their trend in aqueous solubility. As compared with their ligands, the half-sandwich organometallic complexes exhibit remarkably higher aqueous solubility and high solution stability, maintaining stability across diverse biological matrices. These properties are undoubtedly advantageous for *in vivo* applications. The interaction of complexes (**1**) – (**4**) with HSA was studied using CZE and optical spectroscopic methods (spectrofluorimetry and UV–vis spectrophotometry). The complexes bind efficiently to this serum protein, and covalent binding mode can be inferred from measurements with MIM and binding model oligopeptides.

Similar to the ligands, the RhCp* complexes show MDR-selective toxicity potentiated by P-gp in multiple MDR cells. The RuCym complexes are less toxic, demonstrating lower selectivity toward MDR cells. Based on these characteristics, HQCl-pip and its RhCp* complex, as the most promising compounds, were selected to be encapsulated in GM1 and GM3-sph gangliosides, respectively. The nanomicelles showed a high encapsulation efficiency with relatively good stability at pH 7.4. The nanoformulated compounds were tested in a triple coculture of MES-SA, MES-SA/Dx5 and MES-SA/B1 cells, where they exhibited significant MDR-selective activity. Based on its enhanced solubility, stability, and retained MDR-selective toxicity, our results identify the RhCp* complex of HQCl-pip encapsulated in ganglioside-functionalized nanoparticles as an optimal candidate for the pharmacological development of MDR-selective compounds.

## Experimental Section

### Chemicals

All solvents were of analytical grade and used without further purification. [Rh(η^6^-C_5_Me_5_)(μ-Cl)Cl]_2_, [Ru(η^6^-*p*-cymene)(μ-Cl)Cl]_2_, 5-chloro-8-hydroxyquinoline, pyrrolidine, piperidine, aqueous formaldehyde (30% (*v*/*v*)), bpy, *n-*octanol, DMSO-*d*_6_, D_2_O, KH-phthalate, Eagle’s minimum essential medium (EMEM), human serum (from human male AB plasma), HSA (A8763, essentially globulin free), EDTA, DG, 4,4-dimethyl-4-silapentane-1-sulfonic acid (DSS), doxorubicin, 1-methylimidazole (MIM), and human blood serum (H4522, male AB plasma) were Sigma-Aldrich products (St. Louis, MO, USA) and were used without further purification. Terminally protected tripeptides Ac-Ala-His-Ala-NH_2_ (AHA) and Ac-Phe-His-Ala-NH_2_ (FHA) were purchased from GenScript (Piscataway, NJ, USA). NaH_2_PO_4_, Na_2_HPO_4_, KH_2_PO_4_, KCl, KNO_3_, NaCl, HNO_3_, KOH, toluene, methanol, ethanol, CH_2_Cl_2_, diethyl-ether, *n*-hexane were Molar Chemicals (Halásztelek, Hungary) or VWR (Budapest, Hungary) products. Carbocode GmbH (Konstanz, Germany) has patented the synthesis of gangliosides GM1 and GM3-sph and kindly provided these products for our studies.

### Stock Solutions and Sample Preparation

For the preparation of stock and sample solutions, Milli-Q water was used. The aqueous [RhCp*(H_2_O)_3_](NO_3_)_2_ and [RuCym(H_2_O)_3_](NO_3_)_2_ stock solutions were prepared by dissolving an exact amount of the dimeric precursor in water followed by the addition of equivalent amounts of AgNO_3_ and AgCl precipitate was filtered off. The concentrations of the precursor stock solutions were determined by pH-potentiometry using Hyperquad2013^64^ for the calculation. Ligand stock solutions were prepared in an acidic environment (0.01 M nitric acid). Stock solutions of the organometallic complexes were obtained from the ligand and precursor stock solutions by mixing them in a 1:1 concentration ratio, or the isolated complexes were dissolved directly for this purpose. Stock solutions of HSA were prepared in modified PBS buffer (PBS’) containing 12 mM Na_2_HPO_4_, 3 mM KH_2_PO_4_, 1.5 mM KCl and 100.5 mM NaCl; in which the concentration of the components (K^+^, Na^+^, and Cl^–^ ions) corresponds to the human blood serum. Residual citrate content of HSA was removed by repeated ultrafiltration of the protein stock solution, and its concentration was calculated from its UV absorption: *λ*_280 nm_ (HSA) = 36,850 M^–1^ cm^–1^.^65^ HSA containing samples were prepared in PBS’ buffer solutions and were incubated for 24 h at 25 °C. Human serum was diluted with PBS’ buffer four times for stability measurements.

### Synthesis and Characterization of the Ligands and Complexes (**1**–**4**)

#### Synthesis of 5-Chloro-7-(pyrrolidin-1-ylmethyl)quinoline-8-ol (HQCl-Pyr)

0.41 g (2.28 mmol) portion of 5-chloro-8-hydroxyquinoline, 300 μL (2.74 mmol, 1.2 equiv) of 35% formaldehyde solution in H_2_O, and 200 μL (2.44 mmol, 1.07 equiv) of pyrrolidine were dissolved in 20 mL of toluene in a round-bottom flask. The reaction mixture was stirred under reflux for 8 h. The reaction was followed by TLC. After completion the mixture was allowed to cool to room temperature and evaporated to dryness. The crude product was treated with 5 mL ice cold EtOH and the crystals were filtered off and dried to afford a light yellow fine powder. Yield: 468 mg (78%); melting point: 128–130 °C. ESI-MS (methanol, positive): calc. for [M + 1] (C_14_H_16_ClN_2_O): 263.0951 (*m*/*z*) found: 263.0944 (*m*/*z*). ^1^H NMR (DMSO-*d*_6_, δ/ppm, Figure S1): 9.095 (d, *J* = 4.87 Hz, 0.08 H, H(4)), 9.077 (d, *J* = 3.81 Hz, 0.91 H, H(4)), 8.614 (d, *J* = 8.31 Hz, 1H, H(2)), 7.832 (m, *J* = 8.51 Hz; 4.13 Hz, 1H, H(3)), 7.774 (s, 0.94 H, H(6)), 7.676 (s, 0.05 H, H(6)), 4.008 (s, 2H, H(9)), 2.714 (t, *J* = 5.74 Hz, 4H, H(11 and 14)), 1.899 (m, 4H, H(12 and 13)). ^13^C NMR (DMSO-*d*_6_,/ppm, Figure S2): 151.33 (C(8)), 149.41 (C(2)), 139.36 (C(8a)), 132.75 (C(4)), 128.65 (C(6)), 125.30 (C(5)), 123.00 (C(3)), 122.08 (C(4a)), 118.53 (C(7)), 54.22 (C(9)), 53.88 (C(11 and 14)), 23.74 (C(12 and 13)).

#### Synthesis of 5-Chloro-7-(piperidin-1-ylmethyl)quinoline-8-ol (HQCl-Pip)

0.41 g (2.28 mmol) 5-chloro-8-hydroxyquinoline, 300 μL (2.74 mmol, 1.2 equiv) of 35% formaldehyde solution in H_2_O, and 241 μL (2.44 mmol, 1.07 equiv) of piperidine were dissolved in 20 mL of toluene in a round-bottom flask. The reaction mixture was stirred under reflux for 1.5 h. The reaction was followed by TLC. After completion the mixture was allowed to cool to room temperature and evaporated to dryness. The crude product was treated with 5 mL ice cold EtOH and the crystals were filtered and dried to afford a light beige fine powder. Yield: 417 mg (66%); melting point: 118–119 °C. ESI-MS (methanol, positive): calc. for [M + 1] (C_15_H_18_ClN_2_O): 277.1108 (*m*/*z*) found: 277.1101 (*m*/*z*). ^1^H NMR (DMSO-d6, δ/ppm, Figure S3): 9.097 (d, *J* = 4.02 Hz, 0.12 H, H(4)), 9.071 (d, *J* = 3.96 Hz, 0.87 H, H(4)), 8.609 (d, *J* = 8.46 Hz, 1H, H(2)), 7.826 (m, *J* = 8.51 Hz; 4.14 Hz, 1H, H(3)), 7.747 (s, 0.88 H, H(6)), 7.675 (s, 0.10 H, H(6)), 3.873 (s, 2H, H(9)), 2.609 (t, *J* = 4.40 Hz, 4H, H(11 and 15)), 1.695 (m, 4H, H(12 and 14)), 1.578 (m, 2H, H(13)). ^13^C NMR (DMSO-*d*_6_, δ/ppm, Figure S4): 151.87 (C(8)) 149.45 (C(2)), 139.40 (C(8a)), 132.71 (C(4)), 128.64 (C(6)), 125.36 (C(5)), 122.99 (C(3)), 121.13 (C(4a)), 118.54 (C(7)), 57.72 (C(9)), 54.20 (C(11 and 15)), 26.03 (C(12 and 14)), 24.23 (C(13)).

#### Synthesis of [RhCp*(HQCl-Pyr)Cl]Cl (**1**)

HQCl-P5 ligand (5.15 mg, 19.60 μmol) and [Rh(η^5^-C_5_Me_5_)Cl_2_]_2_ (6.00 mg, 9.79 μmol) were dissolved in 10 mL of dichloromethane. The mixture was stirred at room temperature for 24 h, then the solution was evaporated using rotavapor. The solid product then was dissolved in dichloromethane, followed by precipitation using *n*-hexane. The formed orange solid complex was filtrated, washed with *n*-hexane and dried. The same synthetic route was applied to the other complexes. Yield: 7.38 mg (70.3%). ESI-MS (methanol, positive): calc. for [RhCp*(HQCl-pyr)]^2+^ (C_24_H_30_ClN_2_ORh): 250.0545 (*m*/*z*) found: 250.0524 (*m*/*z*). ^1^H NMR (DMSO-*d*_6_, δ/ppm, Figure S5): 10.05 (broaden peak, 1H, H_lig_(N_pyr_H^+^)), 9.094 (d, *J* = 4.19 Hz, 1H, H_lig_(4)), 8.593 (d, *J* = 8.56 Hz, 1H, H_lig_(2)), 7.939 (m, *J* = 8.56 Hz; 4.88 Hz, 1H, H_lig_(3)), 7.824 (s, 1H, H_lig_(6)), 4.539 (md, *J* = 23.75; 12.64; 5.24 Hz, 2H, H_lig_(9)), 3.557–3.396 (Under solvent peak, m, 4H, H_lig_(11 and 14)), 2.196 and 2.028 (m and m, 4H, H_lig_(12 and 13)), 1.824 (s, 15H, H_C5Me5_(CH_3_)). ^13^C NMR (DMSO-*d*_6_, δ/ppm, Figure S6): 152.91 (C(8)), 149.93 (C(2)), 139.18 (C(8a)), 133.26 (C(4)), 130.24 (C(6)), 126.90 (C(5)), 124.42 (C(3)), 119.05 (C(4a)), 114.66 (C(7)), 99.29; 99.23 (C(C_5_)), 53.42 (C(11 and 14)), 51.30 (C(9)), 23.03 (C(12 and 13)), 9.06 (C(Me_5_)).

#### Synthesis of [RuCym(HQCl-Pyr)Cl]Cl (**2**)

HQCl-pyr ligand (4.29 mg, 16.33 μmol), [Ru(η^6^-*p-*cymene)Cl_2_]_2_ (5.00 mg, 8.16 μmol) Yield: 4.58 mg (49.3%). ESI-MS (methanol, positive): calc. for [RuCym(HQCl-pyr)]^2+^ (C_24_H_29_ClN_2_ORu): 249.0506 (*m*/*z*) found: 249.0496 (*m*/*z*); [RuCym(HQCl-pyr)Cl]^+^ (C_24_H_29_Cl_2_N_2_ORu): 533.0695 (*m*/*z*) found: 533.0635 (*m*/*z*).

^1^H NMR (DMSO-*d*_6_, δ/ppm, Figure S7): 9.799 (broaden peak, 1H, H_lig_(N_pyr_H^+^)), 9.543 (d, *J* = 4.64 Hz, 1H, H_lig_(4)), 8.554 (d, *J* = 8.72 Hz, 1H, H_lig_(2)), 7.911 (m, *J* = 8.43 Hz; 5.01 Hz, 1H, H_lig_(3)), 7.774 (s, 1H, H_lig_(6)), 6.040; 5.815 (t; d, *J* = 5.49 Hz, *J* = 5.42 Hz, 4H, H_cym_(C2, C3, C5, C6)), 4.527 (md, *J* = 24.10; 12.66; 5.77; 4.91 Hz, 2H, H_lig_(9)), 3.566–3.309 (Under solvent peak, m, 4H, H_lig_(11 and 14)), 2.873 (m, 1H, H_cym_(C7)), 2.315 (s, 3H, H_cym_(C10)), 2.180; 2.005 (m, 4H, H_lig_(12 and 13)), 1.266 (dd, 6H, *J* = 22.10; *J* = 6.89 Hz, H_cym_(C8, C9)). ^13^C NMR (DMSO-*d*_6_, δ/ppm, Figure S8): 168.25 (C(8)), 151.84 (C(2)), 144.88 (C(8a)), 134.22 (C(4)), 131.83 (C(6)), 127.79 (C(5)), 124.98 (C(3)), 114.94 (C(4a)), 110.39 (C(7)), 101.22 (C(C4)), 98.60 (C(C1)), 83.17; 82.51; 81.45, 80.45 (C(C2, C3, C5, C6)), 53.11; 53.02 (C(11 and 14)), 51.43 (C(9)), 30.91 (C(C7)), 23.07; 23.01 (C(12 and 13)), 22.52; 22.09 (C(C8, C9)), 18.54 (C(C10)).

#### Synthesis of [RhCp*(HQCl-Pip)Cl]Cl (**3**)

HQCl-pip ligand (50.20 mg, 0.1814 mmol), [Rh(η^5^-C_5_Me_5_)Cl_2_]_2_ (56.05 mg, 0.0907 mmol) Yield: 75.20 mg (75.2%). ESI-MS (methanol, positive): calc. for [RhCp*(HQCl-pip)]^2+^ (C_25_H_32_ClN_2_ORh): 257.0629 (*m*/*z*) found: 257.0624 (*m*/*z*). ^1^H NMR (DMSO-*d*_6_, δ/ppm, Figure S9): 9.481 (broaden peak, 1H, H_lig_(N_pip_H^+^)), 9.096 (d, *J* = 3.95 Hz, 1H, H_lig_(4)), 8.592 (d, *J* = 7.56 Hz, 1H, H_lig_(2)), 7.942 (m, *J* = 8.57 Hz; 4.88 Hz, 1H, H_lig_(3)), 7.816 (s, 1H, H_lig_(6)), 4.481 (md, *J* = 33.78; 12.60; 12.78; 4.93; 4.35 Hz, 2H, H_lig_(9)), 3.562; 3.145 (Under solvent peak, dd; m, 4H, H_lig_(11 and 15)), 1.970; 1.799; 1.571 (m, 6H, H_lig_(12–14)), 1.826 (s, 15H, H_C5Me5_(CH_3_)). ^13^C NMR (DMSO-*d*_6_, δ/ppm, Figure S10): 167.54 (C(8)), 149.82; 149.32 (C(2)), 145.58 (C(8a)), 134.46 (C(4)), 132.64 (C(6)) 128.32 (C(5)), 125.34 (C(3)), 113.70 (C(4a)), 109.94 (C(7)), 94.04; 93.97 (C(C_5_)), 54.26 (C(9)), 52.39 (C(11 and 15)), 22.93 (C(12 and 14)), 21.79 (C(13)), 8.97 (C(Me_5_)).

#### Synthesis of [RuCym(HQCl-Pip)Cl]Cl (**4**)

HQCl-pip ligand (4.52 mg, 16.33 μmol), [Ru(η^6^-*p-*cymene)Cl_2_]_2_ (5.00 mg, 8.16 μmol) Yield: 5.37 mg (56.4%). ESI-MS (methanol, positive): calc. for [RuCym(HQCl-pip)]^2+^ (C_25_H_31_ClN_2_ORu): 256.0584 (*m*/*z*) found: 256.0575 (*m*/*z*); [RuCym(HQCl-pip)Cl]^+^ (C_25_H_31_Cl_2_N_2_ORu): 547.0851 (*m*/*z*) found: 547.0799 (*m*/*z*).

^1^H NMR (DMSO-*d*_6_, δ/ppm, Figure S11): 9.547 (d, *J* = 4.74 Hz, 1H, H_lig_(4)), 9.306 (broaden peak, 1H, H_lig_(N_pip_H^+^)), 8.556 (d, *J* = 8.51 Hz, 1H, H_lig_(2)), 7.913 (m, *J* = 8.59 Hz; 4.93 Hz, 1H, H_lig_(3)), 7.772 (s, 1H, H_lig_(6)),6.040; 5.811 (t; t, *J* = 5.50 Hz, *J* = 5.25 Hz, 4H, H_cym_(C2, C3, C5, C6)), 4.454 (md, *J* = 32.56; 12.44; 4.36 Hz, 2H, H_lig_(9)), 3.528 (m, 2H, H_lig_(11 and 15)), 3.163 (m, 1H, H_lig_(11 and 15)), 3.014 (m, 1H, H_lig_(11 and 15)), 2.861 (m, 1H, H_cym_(C7)), 2.313 (s, 3H, H_cym_(C10)), 1.948; 1.855–1.691; 1.545 (m;m;m, 6H, H_lig_ (12–14)), 1.259 (dd, 6H, *J* = 26.56; *J* = 6.89 Hz, H_cym_(C8, C9)). ^13^C NMR (DMSO-*d*_6_, δ/ppm, Figure S12): 168.68 (C(8)), 151.84 (C(2)), 144.82 (C(8a)), 134.12 (C(4)), 132.30 (C(6)), 127.91 (C(5)), 125.01 (C(3)), 113.60 (C(4a)), 110.32 (C(7)), 101.14 (C(C4)), 98.70 (C(C1)) 83.23; 82.52; 81.37, 80.41 (C(C2, C3, C5, C6)), 53.86 (C(9)), 52.36; 52.19 (C(11 and 15)), 30.90 (C(C7)), 22.81; 22.77 (C(12 and 14)), 22.53; 22.06 (C(C8, C9)), 21.75 (C(13)), 18.56 (C(C10)).

### NMR Spectroscopy

Bruker Avance III HD Ascend 500 Plus instrument (Billerica, MA, USA) was used for NMR studies. ^1^H NMR spectroscopic measurements were carried out with a WATERGATE water suppression pulse scheme in the presence of 10% (*v*/*v*) D_2_O in most cases. DSS internal standard was added to samples to obtain reference peaks. ^1^H NMR titrations were carried out in the presence of 0.20 M KNO_3_. The computer program HypSpec^64^ was used to obtain equilibrium constants.

For ligand and complex characterization,^13^C NMR spectra were recorded in DMSO-*d*_6_ (10 mM) with the attached proton test (APT) showing CH and CH_3_ as positive peaks, while quaternary C and CH_2_ appear as negative peaks.

### Electrospray Mass Spectrometry

A Waters Q-TOF Premier (Micromass MS Technologies, Manchester, UK) mass spectrometer with an electrospray ion source was used to perform high-resolution (HR) ESI-MS experiments. Samples contained 100 μM compounds (ligand or complex) in methanol (LC-MS grade).

### Synthesis of Ganglioside-Based Nanomicelles of HQCl-Pip and [RhCp*(HQCl-Pip)Cl]Cl (**3**)

HQCl-pip and its complex (**3**) were entrapped in ganglioside nanomicelles. Spontaneous micelle formation of gangliosides is promoted at a sufficiently high salt concentration. HQCl-pip is more soluble at acidic pH; thus, its nanocarrier, GM1 (100 mg/mL for the size and zeta potential analysis, 50 mg/mL for drug release and cytotoxicity studies), was dissolved in 0.1 M NaCl solution after adjusting the pH to 3 using 1 M HCl solution. HQCl-pip (5.0 mg/mL (18.1 mM) for the size and zeta potential analysis, 2.5 mg/mL (9.03 mM) drug release and cytotoxicity studies) was added to the GM1 solution and stirred overnight. In preliminary experiments, GM3-sph was more efficient for entrapment of (**3**), hence it (100 mg/mL for the size and zeta potential analysis, 50 mg/mL drug release and cytotoxicity studies) was dissolved in 0.1 M NaCl solution, and (**3**) (5.0 mg/mL (8.5 mM) for the size and zeta potential analysis, 2.5 mg/mL (4.3 mM) drug release and cytotoxicity studies) was added to the GM3-sph solution and stirred for 4 h. The formed nanomicelle solutions were dialyzed using a SnakeSkin membrane tube with 3.5 kDa cutoff to remove the free drugs. The drug content was measured both in the dialyzate and retentate by Thermo Scientific Multiscan Sky UV–vis spectrophotometer (Thermo Scientific, US) at 237 nm for (**3**) and 247 nm for HQCl-pip. The EE% is defined as the mass of drug successfully entrapped into the nanomicelles related to the mass of the total drug originally dissolved in percent.

Size distribution of nanomicelles and zeta potential were measured by a Malvern Zetasizer Pro Malvern Instruments, Malvern, UK) using photon correlation spectroscopy. The morphology was examined by using a FEI Talos F200XG2 scanning/transmission electron microscope (S/TEM, Thermo Fischer Scientific, Waltham, MA, USA) after staining the nanomicelles with phosphotungstic acid. The S/TEM imaging was performed by the HUN-REN-PE Environmental Mineralogy Research Group at the University of Pannonia (Veszprém, Hungary).

The *in vitro* drug release test was carried out by a dialysis method in PBS (pH 7.4) and CH_3_COOH/CH_3_COONa buffer (pH 5.5). The samples in dialysis membrane with 3.5 kDa cutoff were incubated for 7 days at 37 °C and shaken at 700 rpm in an environmental incubator shaker (New Brunswick Scientific Co. Inc. G24). Aliquots of both dialyzates were taken at 0, 4, 24, 48, 72, 144, and 168 h and concentration of drugs was determined as described above for drug content.

### pH-Potentiometric Measurements

The pH-potentiometric measurements were carried out at 25.0 ± 0.1 °C in water in the pH range between 2.0 and 11.5 and at a constant ionic strength of 0.20 M KNO_3_. The titrations were performed in a carbonate-free KOH solution (0.20 M). The exact concentrations of the HNO_3_ and KOH solutions were determined by pH-potentiometric titrations. An Orion 710A pH-meter equipped with a Metrohm combined electrode (type 6.0234.100) and a Metrohm 665 Dosimat buret were used for this purpose. The electrode system was calibrated according to the method suggested by Irving et al.^66^ The average water ionization constant, p*K*_w_, was determined as 13.76 ± 0.01, which is in good agreement with the literature data.^67^ The initial volume of the samples was 10.0 mL. Due to the low water solubility of the ligands, experiments could only be utilized for the organometallic complexes, and the concentration was 1.1 mM in all cases. Samples were degassed by bubbling purified argon through them for about 10 min prior to the measurements, and the inert gas was also passed over the solutions during the titrations. The computer program Hyperquad2013^64^ was utilized to establish the stoichiometry of the species and to calculate the equilibrium constants.

### UV–Visible Spectrophotometry and Spectrofluorometry

An Agilent Cary 8454 diode array spectrophotometer was utilized to obtain UV–vis spectra in the wavelength range 190–1100 nm. The path length (*l*) was 1 or 2 cm in most cases (the actual *l* is always indicated in the legends of the figures). The concentrations of the ligands and complexes were between 36 and 100 μM. The ligands were titrated in the presence of 10 equiv of EDTA. Individual samples contained 0–37 μM HSA, 0–206 μM MIM, 0–759 μM AHA, or 0–733 μM FHA. Spectra were always background and baseline corrected. The computer program HypSpec^64^ was used to obtain stability constants.

Fluorescence measurements were carried out on a Fluoromax (Horiba Jobin Yvon, Longjumeau, France) spectrofluorometer using a 1 cm × 1 cm quartz cuvette. Samples contained 1 μM HSA or 1 μM DG and 1 μM HSA and the complex concentration was varied between 0 and 50 μM. Spectroscopic measurements were carried out on individually prepared samples. Excitation wavelengths were 295 nm for Trp-214 quenching and 340 nm for the DG displacement studies. The calculated conditional stability constants for HSA–complex species were obtained using the computer program HypSpec.^64^ Calculations always were based on data obtained from at least two independent measurements. Self-absorbance and inner filter effect had to be taken into account,^68^ and corrections were made as it was described in our former works.^53,54^

### Determination of Distribution Coefficients

The traditional shake-flask method was used to obtain distribution coefficients of the ligands as well as of the complexes in *n*-octanol/buffered aqueous solution (20 mM phosphate, pH = 7.4) at different chloride ion concentrations (4, 24, and 100 mM) using UV–vis spectrophotometry (Agilent Cary 8454 diode array spectrophotometer, Santa Clara, CA, USA) for the analysis. The compounds were dissolved in buffered aqueous solutions previously saturated with *n*-octanol. Then the aqueous and *n*-octanol phases were gently mixed in different volume ratios (using 1:60 for the ligands, 1:1 for the RhCp* and 1:2 for the RuCym complexes ratios of *n*-octanol to buffered aqueous solution volume) for 4 h, followed by phase separation. Then the UV–vis spectrum of the aqueous or *n*-octanol phase was recorded and compared to the reference spectrum. Stock solutions were made in the aqueous phase, and distribution coefficients (D_pH_) were calculated by the following equation:
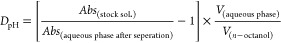


### Determination of Aqueous Thermodynamic Solubility at pH = 7.4 (*S*_7.4_)

Thermodynamic solubility (*S*_7.4_) of the ligands was assessed by measuring the saturation levels in water at pH = 7.4 (10 mM HEPES buffer) and 25.0 ± 0.1 °C. The concentration of the compounds was determined by UV–vis spectrophotometry. For calibration, stock solutions of the compounds were used with known concentrations dissolved in 100% DMSO, 75% DMSO, and 50% (*v*/*v*) DMSO/buffered aqueous solutions.

### Capillary Zone Electrophoresis

An Agilent 7100 capillary electrophoresis system equipped with a diode-array UV–vis detector (200–600 nm) was utilized to gain electropherograms. Fused silica capillaries of 48 cm total length with 75 μm inner diameters were used (Agilent Technologies, Santa Clara, CA, USA). The background electrolyte (BGE) was PBS’ buffer (pH = 7.4), in which the samples were also made. The conditioning process of new capillaries and daily preparation were performed as described formerly.^54^ In order to ensure a steady baseline, the capillary was flushed with BGE (2 min) before each run and was rinsed with NaOH (0.1 M; 1.5 min), H_2_O (1.5 min), and then with BGE (2 min) after each run. As postconditioning, the capillary was also flushed with BGE for 1 min. The sample tray and the capillary cassette were kept at 25 °C. The hydrodynamic injection was used at 50 or 100 mbar for a 5 s injection time. For separation, 25 kV voltage and 140 μA current were applied; however, only ca. 20 kV voltage could be reached. The sample run time was set to 6 min. The computer program ChemStation (Agilent) was used to record electropherograms.^69^ Electropherograms and spectra of the corresponding peaks were also collected. In order to check the purity of the isolated organometallic complexes, measurements were performed at 50–100 μM compound concentration with the corresponding ligands and precursors as well.

Interaction of the organometallic complexes with HSA was studied at constant protein concentration (20 μM) in PBS’ buffer (pH = 7.4), and the HSA-to-complex ratio varied between 0.03 and 0.60.

### Crystallization, X-Ray Data Collection, Structure Solution, and Refinement for Complex (**3**)

Single crystal of [RhCp*(HQCl-pip)Cl]Cl·H_2_O·OC_4_ was obtained from dichloromethane solution with diethyl-ether by vapor diffusion method. Yellow, platelet crystal was mounted on a loop and transferred to the goniometer. X-ray diffraction data were collected at room temperature (293(2) K) on a SuperNova, Dual, Cu at home/near diffractometer using a CCD plate detector and Cu Kα radiation. Multiscan absorption correction was carried out using the program CrysAlisPro^70^ Olex2^71^ software was used for structure solution and refinement using the SHELX^72^ program package, which was done by full-matrix least-squares on F^2^. Refinement of non-hydrogen atoms was carried out with anisotropic temperature factors. Hydrogen atoms were placed in geometric positions. They were included in structure factor calculations, but they were not refined. The isotropic displacement parameters of the hydrogen atoms were approximated from the U(eq) value of the atom to which they were bonded to. The diethyl ether molecule was found in two disordered positions at 50–50% due to which the uncertainty of the O–C and C–C distances is large, and no hydrogen atoms were fitted for this molecule. The summary of data collection and refinement parameters are collected in Table S1. Selected bond lengths and angles of compounds were calculated by PLATON software.^73^ Graphical representation and the edition of CIF files were done by Mercury^74^ and Encipher^75^ softwares. The crystallographic data files for complex (**3**) have been deposited with the Cambridge Crystallographic Database as CCDC 2382816.

### *In vitro* Cell Studies: Cell Lines, Culture Conditions, and Cytotoxicity Assay

Cell lines were purchased from the American Type Culture Collection (ATCC). MES-SA/B1 is a human MDR1-transfected derivative of MES-SA, and MES-SA mCherry, MES-SA/B1 mOrange, and MES-SA/Dx5 eGFP were engineered to stably express the respective fluorescent proteins previously.^62^ Cells were kept in culture medium (DMEM) supplemented with 10% fetal bovine serum, 2 mM l-glutamine, 100 units/mL penicillin, and 100 μg/mL streptomycin (Thermo Fisher). One week before use, MES-SA/Dx5 eGFP cells were treated with 0.5 μM doxorubicin to ensure ABCB1/P-gp expression.

The tested compounds were dissolved in 50% (*v*/*v*) DMSO/PBS’ buffer to prepare 5–10 mM stock solutions, which were diluted in complete culture medium. Constitutive expression of the fluorescent proteins allowed multiplexing in cytotoxicity experiments.^62^ Cells were mixed before seeding in 384-well plates: in double cocultures, MES-SA mCherry and MES-SA/Dx5 eGFP cells were seeded at 1250–1250 cells/well (in total 2500 cells/well); while in triple cocultures, MES-SA mCherry, MES-SA/Dx5 eGFP and MES-SA/B1 mOrange cells were seeded at 800–800–800 cells/well (in total 2400 cells/well). Serial dilutions of the compounds were added 24 h after seeding. To address the effect of P-gp function, TQ was added at a nontoxic concentration (0.4 μM) 15 min before the test compounds. The intensity of the fluorescent proteins, which is proportional to cell mass, was measured after 144 h of incubation at the respective fluorescent channels (excitation/emission: eGFP: 485 nm/510 nm; mOrange: 545 nm/567 nm; mCherry: 585 nm/610 nm). pIC_50_ values were calculated by a custom program written by Judit Sessler in C#.^62^ IC_50_ values and standard deviation were calculated from average pIC_50_ values from at least 3 independent measurements.

HQCl-pip and HQCl-pyr were also tested on the human Colo205 (chemosensitive, ATCC-CCL-222) and Colo320 (Pgp/MDR1-expressing, doxorubicin-resistant, ATCC-CCL-220.1) colon adenocarcinoma cell lines following the protocol described previously.^14^

### Antibacterial Effect: Bacterial Culture and Determination of MIC Values

*Escherichia coli* (ATCC 25922) was used as Gram-negative strain and *Staphylococcus aureus* (ATCC 25923, methicillin-resistant (MRSA) ATCC 43300) strains were applied as Gram-positive bacterial cultures in the experiments. MIC values of the compounds were determined in 96-well plates based on the Clinical and Laboratory Standard Institute guidelines (CLSI guidelines).^76^ The stock solutions of the compounds (same as those used for cytotoxicity experiments) were diluted in 100 μL of Mueller Hinton Broth. Then, 10^–4^ dilution of an overnight bacterial culture in 100 μL of medium was added to each well, with the exception of the medium control wells. The plates were further incubated at 37 °C for 18 h; at the end of the incubation period, the MIC values of the tested compounds were determined by visual inspection.

## Supporting Information Available

^1^H and ^13^C NMR spectra of the compounds; crystallographic data for [RhCp*(HQCl-pip)Cl]Cl·H_2_O·OC_4_; additional pH-dependent ^1^H NMR and UV–vis spectra of the complexes; UV–vis spectra of the complexes in the presence of various equivalents of 2,2′-bipyridine, chloride ions, HSA, and MIM; and cytotoxicity and antibacterial data (PDF)

## Abbreviations

RhCp*: Rh(η^5^-C_5_Me_5_)
RuCym: Ru(η^6^-*p*-cymene)
HQCl-pyr: 5-chloro-7-(pyrrolidin-1-ylmethyl)quinolin-8-ol
HQCl-pip: 5-chloro-7-(piperidin-1-ylmethyl)quinolin-8-ol
ATP: adenosine triphosphate
HQ: 8-hydroxyquinoline
p*K*_a_: proton dissociation constant
SR: selectivity ratio
MDR: multidrug resistance
ESI-MS: electrospray ionization mass spectrometry
CZE: capillary zone lectrophoresis
NMR: nuclear magnetic resonance
GM: ganglioside
HSA: human serum albumin
MIC: minimum inhibitory concentration
P-gp: P-glycoprotein
ABC: ATP-binding cassette
ORTEP: oak ridge thermal-ellipsoid plot program
HQCl-D-hPro: (*R*)-5-chloro-7-((homoproline-1-yl)methyl)8-hydroxyquinoline
UV–vis: UV–visible
EDTA: ethylenediaminetetraacetic acid
PHQ: 7-(1-piperidinylmethyl)quinolin-8-ol
HQCl-d-Pro: (*R*)-5-chloro-7-((proline-1-yl)methyl)8-hydroxyquinoline
EMEM: Eagle’s minimum essential medium
His: histidine
MIM: 1-methylimidazole
AHA: Ac-Ala-His-Ala-NH_2_
FHA: Ac-Phe-His-Ala-NH_2_
Trp: tryptophan
DG: dansylglycine
sph: sphingosine
EE%: encapsulation efficiency
S/TEM: scanning/transmission electron microscopy
TQ: tariquidar
MRSA: methicillin-resistant *S. aureus*
TLC: thin layer chromatography
APT: attached proton test
HR: high-resolution
p*K*_w_: water ionization constant
BGE: background electrolyte
bpy: 2,2′-bipyridine
DSS: sodium trimethylsilylpropanesulfonate
PBS’: phosphate-buffered saline buffer
ATCC: American Type Culture Collection
CLSI: Clinical and Laboratory Standard Institute
