# Supplementary material for: Organometallic Half-Sandwich Complexes of 8-Hydroxyquinoline-Derived Mannich Bases with Enhanced Solubility: Targeting Multidrug Resistant Cancer
Source: Inorg Chem. 2024 Dec 5;63(50):23983–98. doi: 10.1021/acs.inorgchem.4c04398 (PMC11653257; doi:10.1021/acs.inorgchem.4c04398)
Supplement: Supplementary file 1 — ic4c04398_si_001.pdf [file ic4c04398_si_001.pdf]

Supplementary Information

Organometallic half-sandwich complexes of 8-hydroxyquinoline derived Mannich bases with enhanced solubility: targeting multidrug resistant cancer

Tamás Pivarcsik,[a,b] Szilárd Tóth,[c,d] Szonja P. Pósa,[c] Nóra V. May,[e] Éva Kováts,[f] Gabriella Spengler,[a,g] Izolda Kántor,[h] Alexandra Rolya,[i] Tivadar Feczkó,[h,i], István Szatmári,[j] Gergely Szakács,[c,k] Éva A. Enyedy[a,b*]

[a] *MTA-SZTE Lendület Functional Metal Complexes Research Group, University of Szeged, Dóm tér 7-8, H-6720 Szeged, Hungary*

[b] *Department of Molecular and Analytical Chemistry, Interdisciplinary Excellence Centre, University of Szeged, Dóm tér 7-8, H-6720 Szeged, Hungary*

[c] *Drug Resistance Research Group, Institute of Molecular Life Sciences, HUN-REN Research Centre for Natural Sciences, Magyar Tudósok krt. 2, H-1117 Budapest, Hungary*

[d] *National Laboratory for Drug Research and Development, Magyar Tudósok krt. 2, H-1117 Budapest, Hungary*

[e] *Centre for Structural Science, HUN-REN Research Centre for Natural Sciences, Magyar Tudósok krt. 2, H-1117 Budapest, Hungary*

[f] *Institute for Solid State Physics and Optics, HUN-REN Wigner Research Centre for Physics, P.O. Box 49, Budapest H-1525, Hungary*

[g] *Department of Medical Microbiology, Albert Szent-Györgyi Health Center and Albert Szent-Györgyi Medical School, University of Szeged, Semmelweis u. 6, H-6725 Szeged, Hungary*

[h] *Institute of Materials and Environmental Chemistry, HUN-REN Research Centre for Natural Sciences, Magyar Tudósok krt. 2, H-1117 Budapest, Hungary*

[i] *Faculty of Engineering, University of Pannonia, Egyetem u. 10, H-8200 Veszprém, Hungary*

[j] *Institute of Pharmaceutical Chemistry, HUN-REN-SZTE Stereochemistry Research Group, University of Szeged, Eötvös u. 6, H-6720 Szeged, Hungary*

[k] *Center for Cancer Research, Medical University of Vienna, Borschkegasse 8a, A-1090 Vienna, Austria*

Table of contents

Synthetic steps of the ligands HQCl-pyr and HQCl-pip SI-3

^{1}H and ^{13}C NMR spectra of the ligands HQCl-pyr and HQCl-pip SI-3

^{1}H and ^{13}C NMR spectra of the complexes **1**–**4** SI-5

Supplementary Information

Electropherogram of the isolated [RhCp*(HQCl-pip)Cl]Cl (**3**) complex SI-9

Crystallographic data of the complex [RhCp*(HQCl-pip)Cl]Cl·H_2O·OC_4 .... SI-10

pH-dependent ^{1}H NMR and UV-vis spectra of HQCl-pyr and HQCl-pip SI-14

Deprotonation processes of the two 8-hydroxyquinoline derived Mannich bases SI-16

UV-vis spectra of the complexes in PBS' at 1 min and after 48 h SI-16

pH-dependent UV-vis spectra of the RhCp* – HQCl-pyr and the RuCym – HQCl-pip systems SI-17

Physico-chemical parameters of the RhCp* and RuCym complexes SI-18

pH-dependent ^{1}H NMR spectra of RhCp*–HQCl-pip SI-19

UV-vis spectra of RhCp*complex of HQCl-pyr at various equivalents of 2,2'-bipyridine SI-20

Time dependent UV-vis spectra of RuCym–HQCl-pyr at 2 equivalents of 2,2'-bipyridine SI-20

UV-vis spectra of RuCym complex of HQCl-pip at various equivalents of chloride ions SI-21

Log$D_{7.4}$ values of the complexes at pH 7.4 .... SI-21

UV-vis and ^{1}H NMR spectra of complex **3** in EMEM followed over time SI-22

UV-vis spectra of RuCym–HQCl-pip at various equivalents of HSA SI-22

UV-vis spectra of RuCym–HQCl-pip at various equivalents of MIM SI-23

Fluoresence emission spectra of HSA at various amount of RhCp*–HQCl-pip SI-23

Cytotoxicity data of HQCl-pyr and HQCl-pip SI-24

Antibacterial activity of the studied compounds SI-25

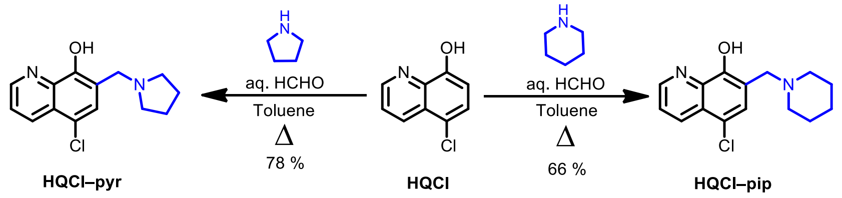

Scheme S1. Synthesis of the 8-hydroxyquinoline derived Mannich bases HQCl-pyr and HQCl-pip.

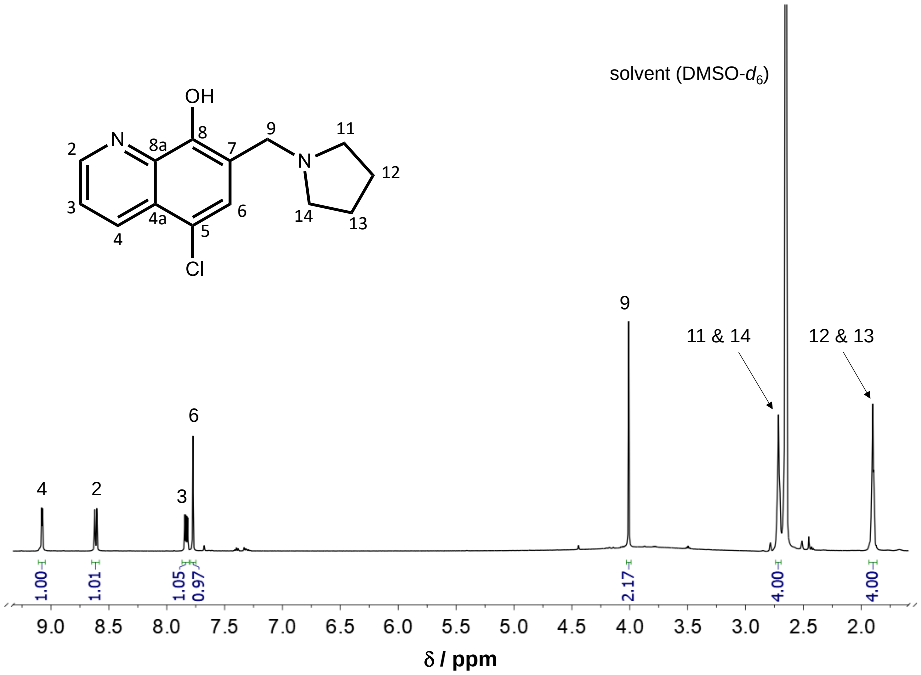

Figure S1. ^{1}H NMR spectrum of HQCl-pyr in DMSO-d_6. The chemical structure shows numbering of peaks. {$c_{\text{HQCl-pyr}}$ = 10 mM, T = 25.0 °C}

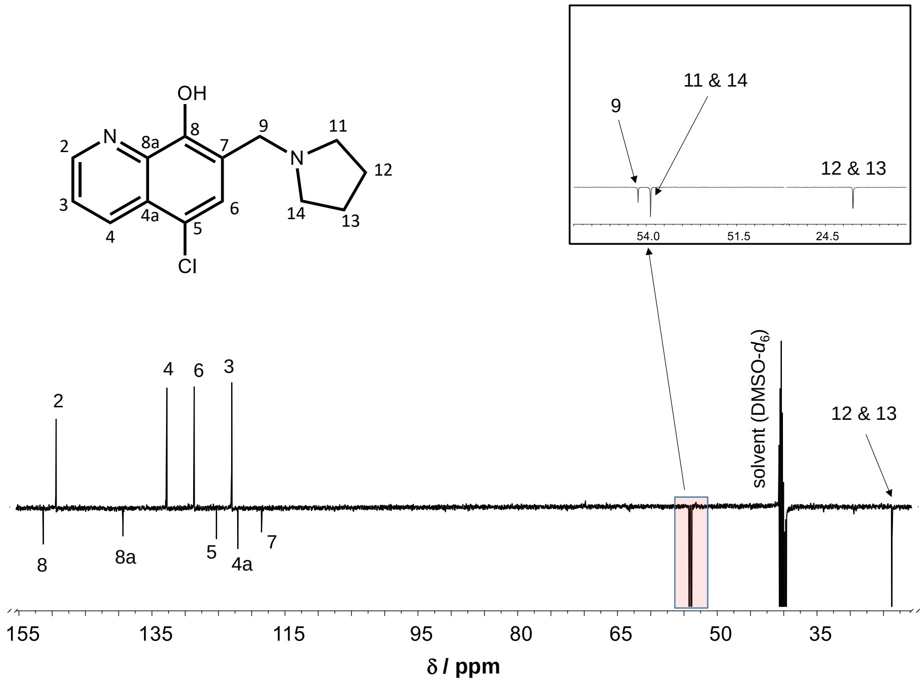

Figure S2. ^{13}C APT NMR spectrum of HQCl-pyr in DMSO-d_6. Attached proton test method: CH and CH_3 peaks are positive, C and CH_2 peaks are negative. The chemical structure shows numbering of peaks. {$c_{\text{HQCl-pyr}}$ = 10 mM, T = 25.0 °C}

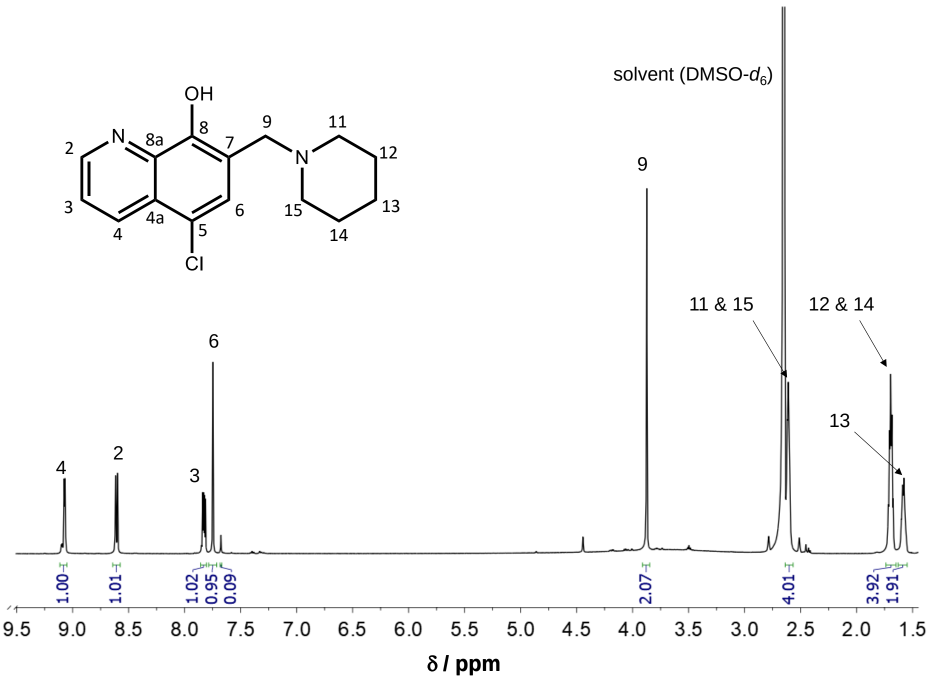

Figure S3. ^{1}H NMR spectrum of HQCl-pip in DMSO-d_6. The chemical structure shows numbering of peaks. {$c_{\text{HQCl-pip}}$ = 10 mM, T = 25.0 °C}

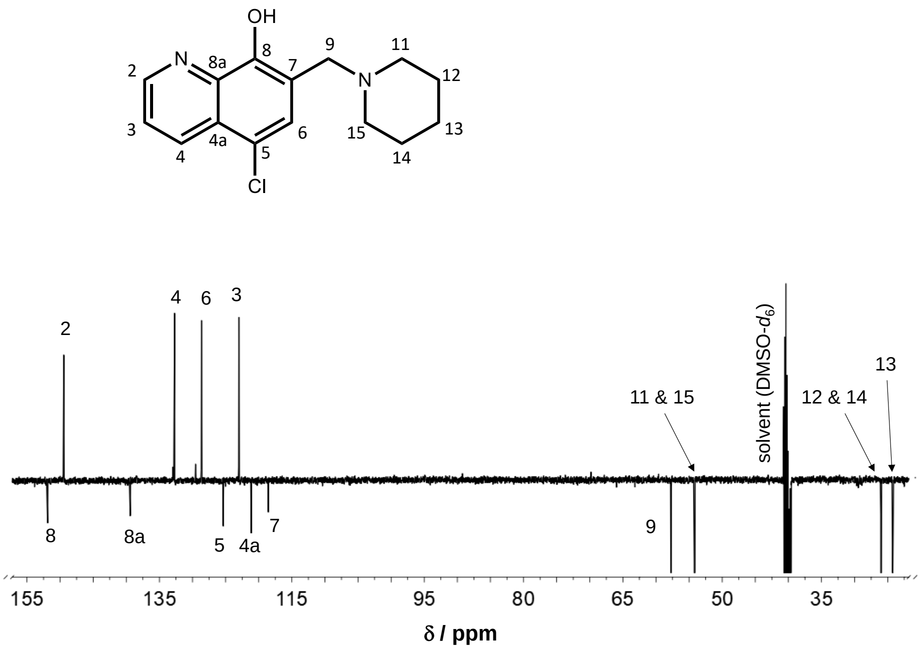

Figure S4. ^{13}C APT NMR spectrum of HQCl-pip in DMSO-d_6. Attached proton test method: CH and CH_3 peaks are positive, C and CH_2 peaks are negative. The chemical structure shows numbering of peaks. {$c_{\text{HQCl-pip}}$ = 10 mM, T = 25.0 °C}

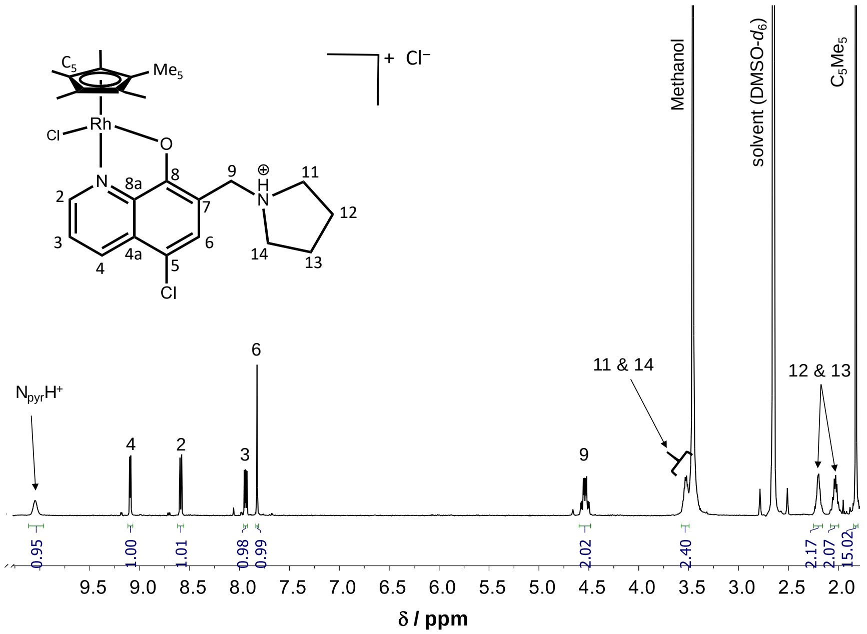

Figure S5. ^{1}H NMR spectrum of [Rh(η^5-C_5Me_5)(HQCl-pyr)Cl]Cl (**1**) in DMSO-d_6. The chemical structure shows numbering of peaks. {c_{complex}= 10 mM, T = 25.0 °C}

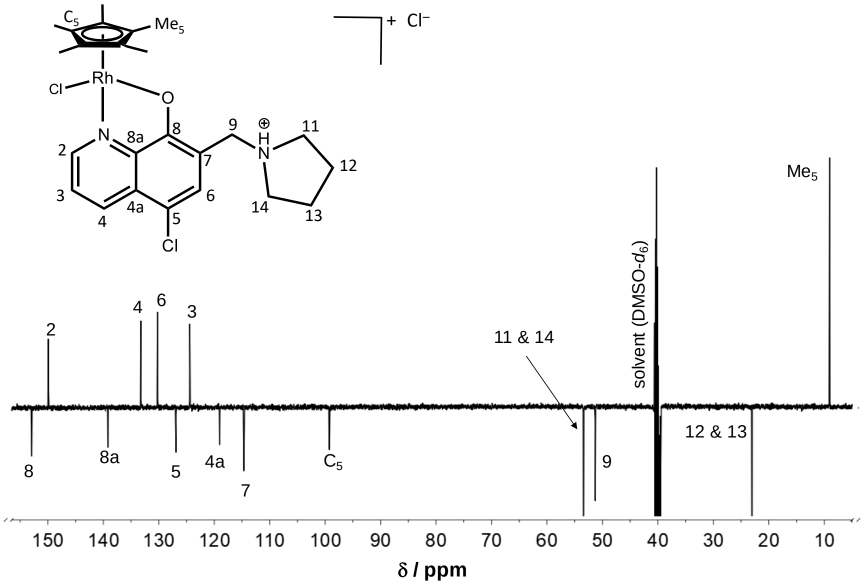

Figure S6. ^{13}C APT NMR spectrum of [Rh(η^5-C_5Me_5)(HQCl-pyr)Cl]Cl (**1**) in DMSO-d_6. Attached proton test method: CH and CH_3 peaks are positive, C and CH_2 peaks are negative. The chemical structure shows numbering of peaks. {$c_{complex}$ = 10 mM, T = 25.0 °C}

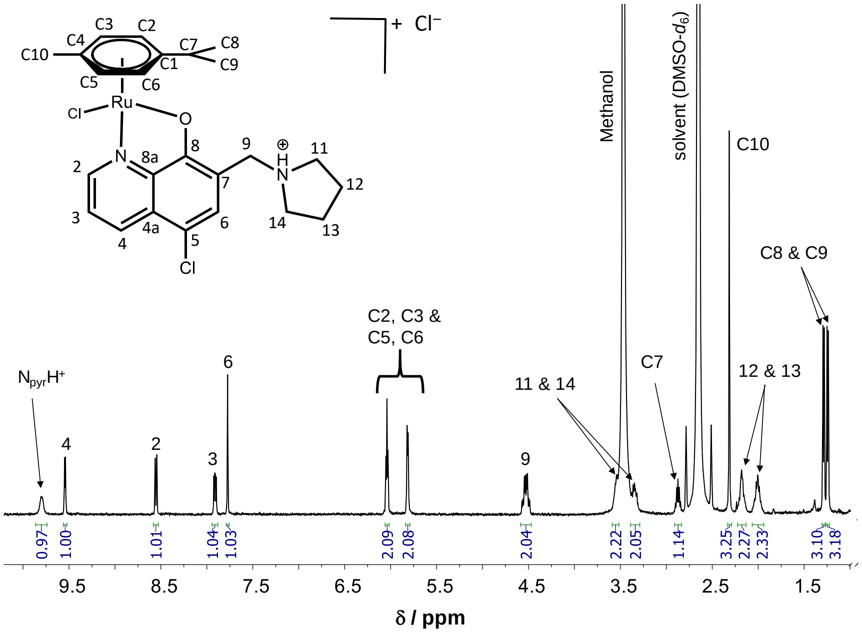

Figure S7. ^1H NMR spectrum of [Ru(η^6-*p*-cymene)(HQCl-pyr)Cl]Cl (**2**) in DMSO-d_6. The chemical structure shows numbering of peaks. {$c_{complex}$= 1 mM, T = 25.0 °C}

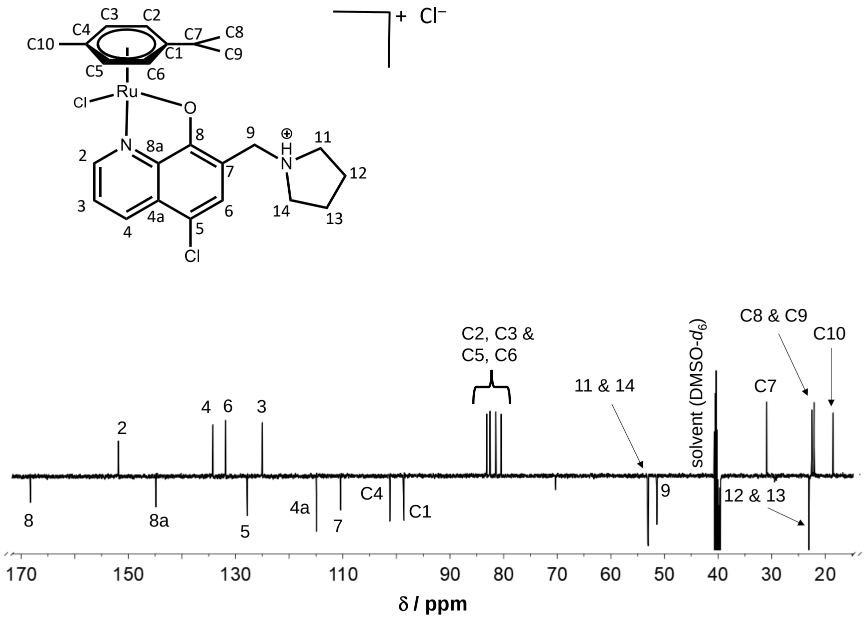

Figure S8. ^{13}C APT NMR spectrum of [Ru(η^6-*p*-cymene)(HQCl-pyr)Cl]Cl (**2**) in DMSO-d_6. Attached proton test method: CH and CH_3 peaks are positive, C and CH_2 peaks are negative. The chemical structure shows numbering of peaks. {c_{complex} = 10 mM, T = 25.0 °C}

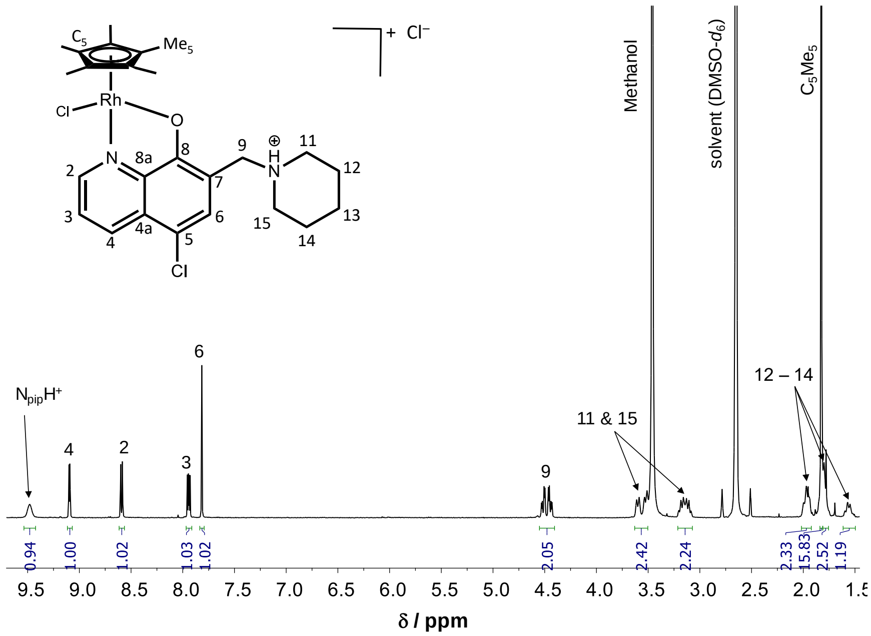

Figure S9. ^{1}H NMR spectrum of [Rh(η^5-C_5Me_5)(HQCl-pip)Cl]Cl (**3**) in DMSO-d_6. The chemical structure shows numbering of peaks. {c_{complex}= 10 mM, T = 25.0 °C}

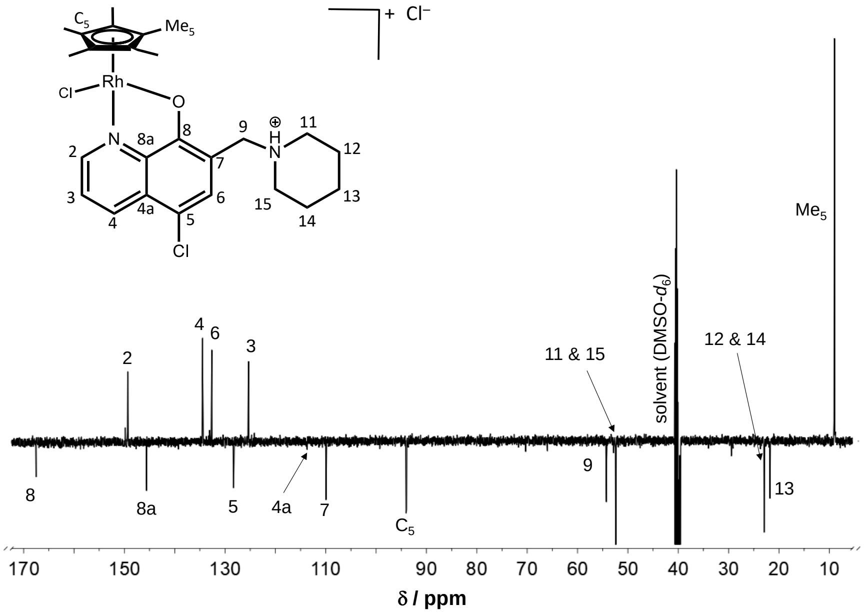

Figure S10. ${}^{13}C$ APT NMR spectrum of [Rh(η^5-C_5Me_5)(HQCl-pip)Cl]Cl (**3**) in DMSO-d_6. Attached proton test method: CH and CH_3 peaks are positive, C and CH_2 peaks are negative. The chemical structure shows numbering of peaks. {$c_{complex}$ = 10 mM, T = 25.0 °C}

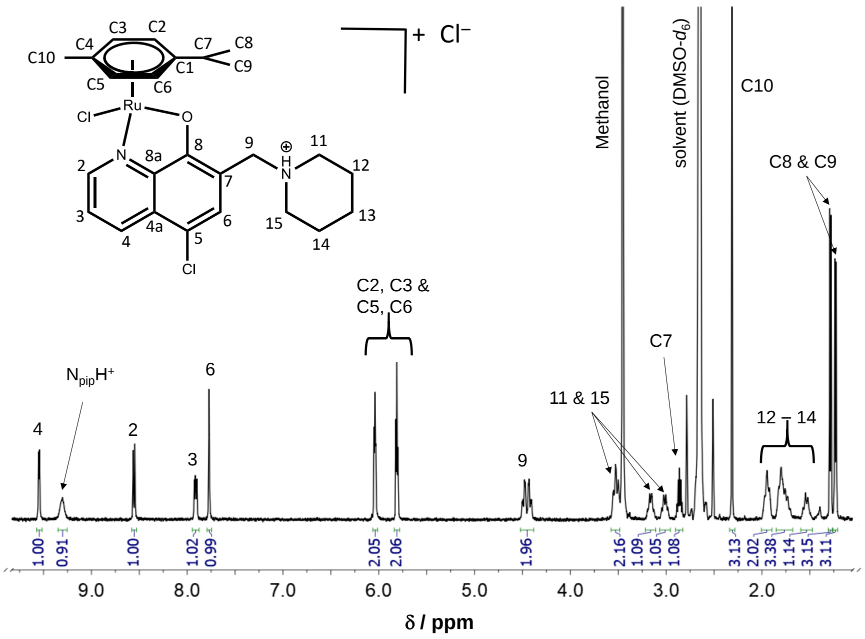

Figure S11. ${}^{1}H$ NMR spectrum of [Ru(η^6-*p*-cymene)(HQCl-pip)Cl]Cl (**4**) in DMSO-d_6. The chemical structure shows numbering of peaks. {$c_{complex}$= 1 mM, T = 25.0 °C}

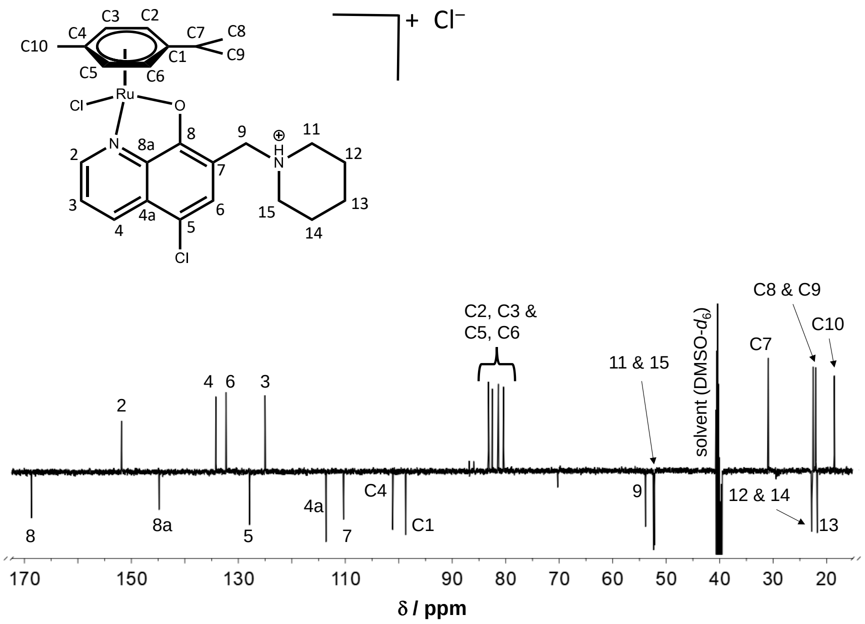

Figure S12. ^{13}C APT NMR spectrum of [Ru(η^6-*p*-cymene)(HQCl-pip)Cl]Cl (**4**) in DMSO-d_6. Attached proton test method: CH and CH_3 peaks are positive, C and CH_2 peaks are negative. The chemical structure shows numbering of peaks. {$c_{complex}$ = 10 mM, T = 25.0 °C}

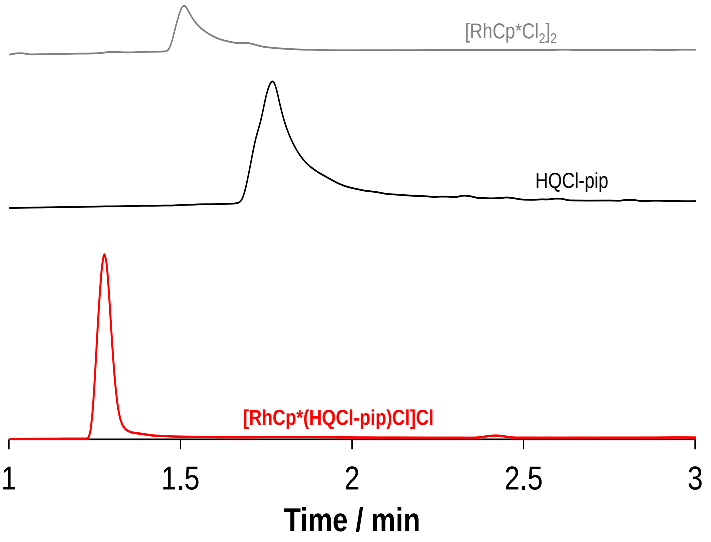

Figure S13. Electropherograms of the isolated [RhCp*(HQCl-pip)Cl]Cl (**3**) complex, HQCl-pip and metal precursor $[RhCp^*Cl_2]_2$. {c = 50 - 100 μM; pH = 7.4 (PBS'); λ = 200 nm}

Supplementary Information

Table S1. Crystal data and structure refinement for the crystal [RhCp*(HQCl-pip)Cl]Cl×H_2O×OC_4

Empirical formula	$C_{52}H_{68}Cl_6N_4O_5Rh_2$
Moiety formula	2($C_{25}H_{32}Cl_2N_2ORh$), 0.5(C_4O), 2(Cl), 2(H_2O)
Formula weight	1247.62
Temperature (K)	293(2)
Radiation and wavelength (Å)	Cu-Kα, λ =1.54184Å
Crystal system	orthorhombic
Space group	$P2_12_12_1$
Unit cell dimensions	
*a (*Å)	8.1963(2)
b (Å)	14.6315(3)
c (Å)	22.9770(5)
α (°)	α = 90
β (°)	β = 90
γ (°)	γ = 90
Volume (Å^3)	2755.50(11)
Z/Z'	2/0.5
Density (calculated) (Mg/m^3)	1.504
Absorption coefficient, μ (mm^{-1})	7.909
F(000)	1280
Crystal colour	yellow
Crystal description	platelet
Crystal size (mm)	0.12 x 0.1 x 0.02
Absorption correction	multi-scan
Max. and min. transmission	0.54332, 1.00000
θ–range for data collection (°)	3.581 ≤ θ ≤7 6.597
Index ranges	-9 ≤ *h* ≤ 10; -17≤ *k* ≤18; -21 ≤ *l* ≤ 28
Reflections collected	22364
Completeness to 2θ	1.000
Independent reflections (R_{int})	5725 (0.0642)
Reflections I>2σ(I)	4607
Refinement method	full-matrix least-squares on F^2
Data / restraints / parameters	5725 /3 /339
Goodness-of-fit on *F*2	1.033
Final *R* indices [I>2σ(I)] (R1, wR2)	0.0377, 0.0885
R indices (all data) (R1, wR2)	0.0539, 0.0969
Max. and mean shift/esd	0.001; 0.000
Largest diff. peak and hole (e.Å^{-3})	0.485; -0.335

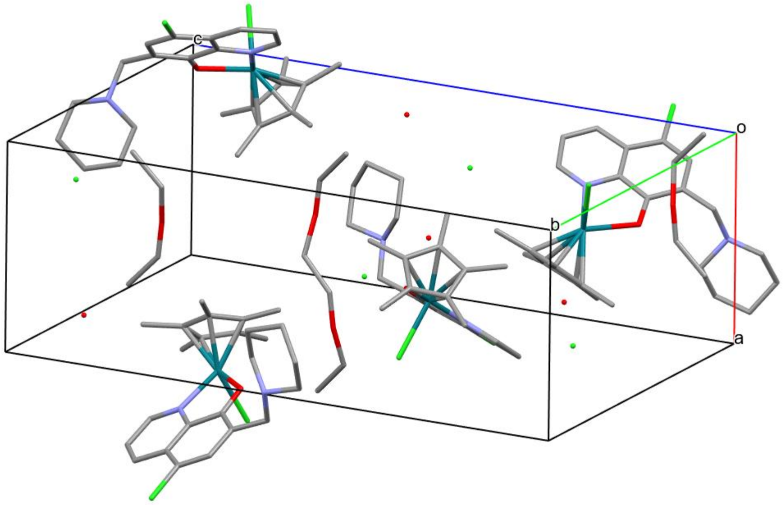

Figure S14. Unit cell of crystal [RhCp*(HQCl-pip)Cl]Cl×H_2O×OC_4

Table S2. Selected bond lengths (Å) and angles (°) for crystal [RhCp*(HQCl-pip)Cl]Cl×H_2O×OC_4

Bond length (Å)	
Rh1-N1	2.103(5)
Rh1-O1	2.105(4)
Rh1-Cl2	2.417(2)
Rh1-C16	2.152(7)
Rh1-C17	2.139(7)
Rh1-C18	2.117(7)
Rh1-C19	2.160(6)
Rh1-C20	2.155(6)
Rh1-Cg(Cp*)	1.773(4)
Bond angles (^{o})	
N1-Rh1-O1	78.5(2)
O1-Rh1-Cl2	87.9(2)
N1-Rh1-Cl2	85.8(2)
Cg(Cp*)-Rh1-O1	127.94(18)
Cg(Cp*)-Rh1-N1	133.63(19)
Cg(Cp*)-Rh1-Cl2	126.49(14)

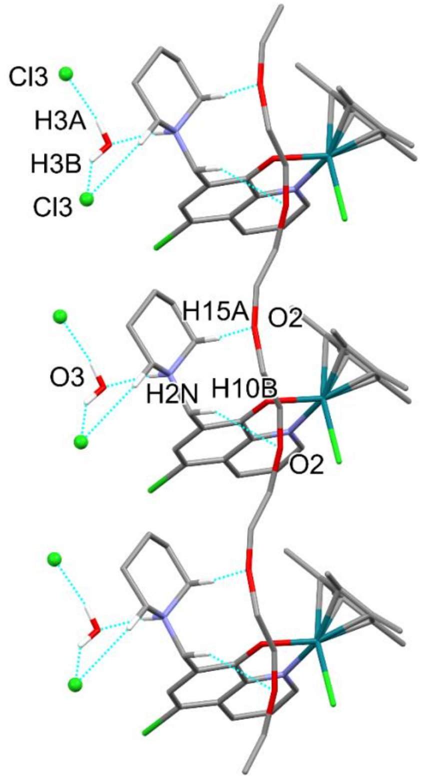

Figure S15. Intermolecular H-bonds in crystal [RhCp*(HQCl-pip)Cl]Cl×H_2O×OC_4

Table S3. Hydrogen-bond geometry of [RhCp*(HQCl-pip)Cl]Cl×H_2O×OC_4

**D-H…A**	**D-H** (Å)	**H…A**(Å)	**D…A**(Å)	**D-H…A** (^{o})	**symmetry codes**
N2-H2N…O3	0.82	1.95	2.757(9)	169	
O3-H3A…Cl3	0.84(5)	2.42(7)	3.113(9)	140(8)	1-x,-1/2+y,1/2-z
O3-H3B…Cl3	0.85(11)	2.42(11)	3.099(8)	137(12)	3/2-x,1-y,1/2+z
C3-H3…Cl1	0.93	2.74	3.103(8)	104	intra
C10-H10B…O1	0.97	2.53	2.892(8)	102	intra
C11-H11A…Cl3	0.97	2.79	3.615(10)	144	3/2-x,1-y,1/2+z
C15-H15A…O2	0.97	2.67	3.582(10)	156	
C15-H10B…O2	0.97	2.76	3.602(10)	146	

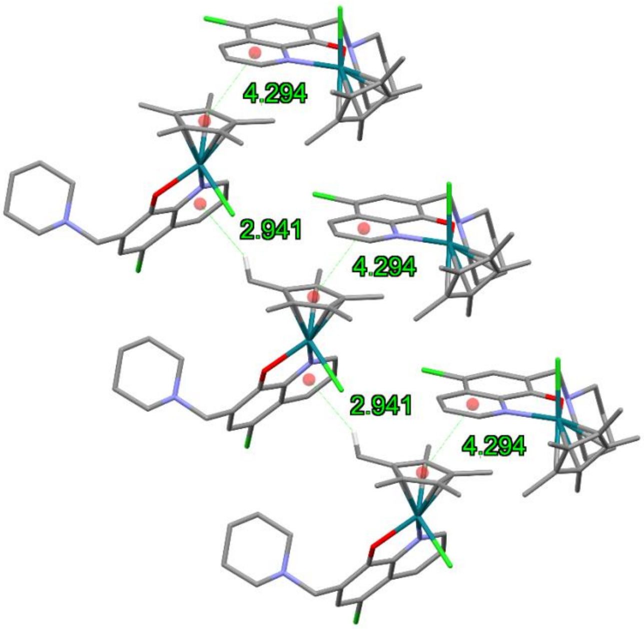

Figure S16. Packing arrangements viewed from the 'ab' plane of crystal [RhCp*(HQCl-pip)Cl]Cl×H_2O×OC_4 showing the intermolecular π … π and C-H… π interactions and the distance values in Å units.

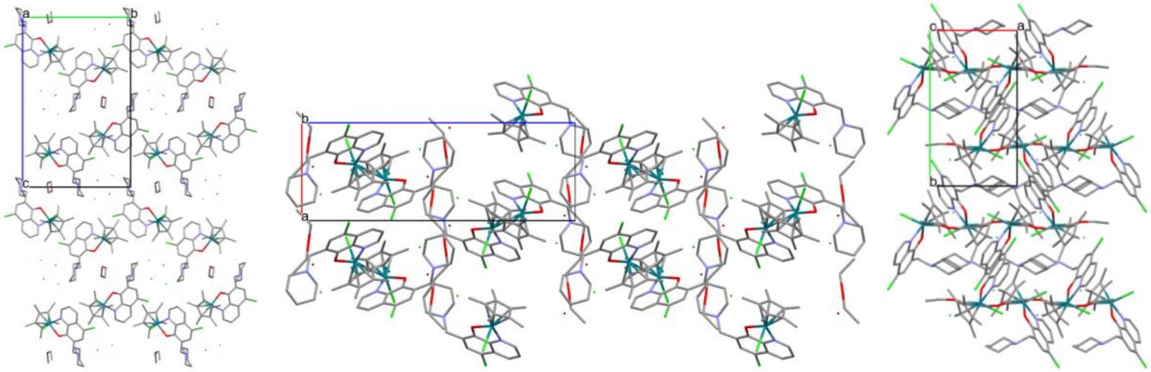

Figure S17. Packing arrangements in crystal [RhCp*(HQCl-pip)Cl]Cl×H_2O×OC_4 viewed from the crystallographic directions 'a', 'b' and 'c'.

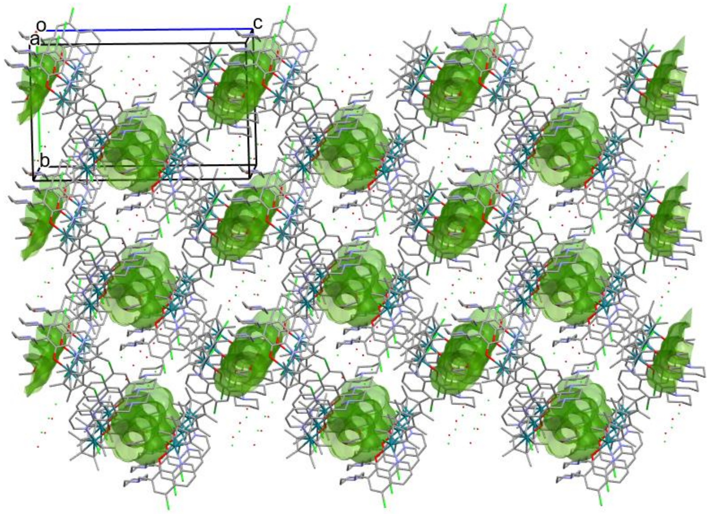

Figure S18. Packing arrangement in crystal [RhCp*(HQCl-pip)Cl]Cl×H_2O×OC_4 showing the channels in green, which contains the disordered diethyl-ether molecules along the *a* crystallographic axis. The volume of the channel is 204 Å^3 which is 7.4% of the unit cell.

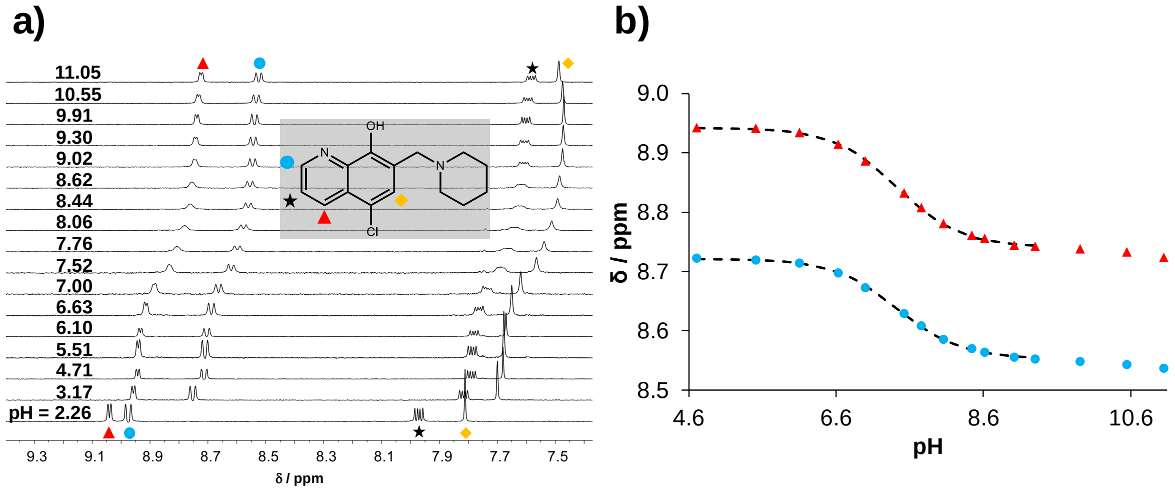

Figure S19. (a) ^{1}H NMR spectra in the low-field region of HQCl-pip at various pH values and (b) chemical shifts (▲ (red) and ● (blue) denotes C^{4}H and C^{2}H protons, respectively) as a function of pH along with the fitted (dashed) lines. The chemical shift values are only fitted in the pH range 4.6 – 9.0 to determine pK_a (OH). {$c_{\text{HQCl-pip}}$ = 400 µM; 10% (*v*/*v*) D_2O; *I* = 0.20 M KNO_3; *T* = 25.0 °C}

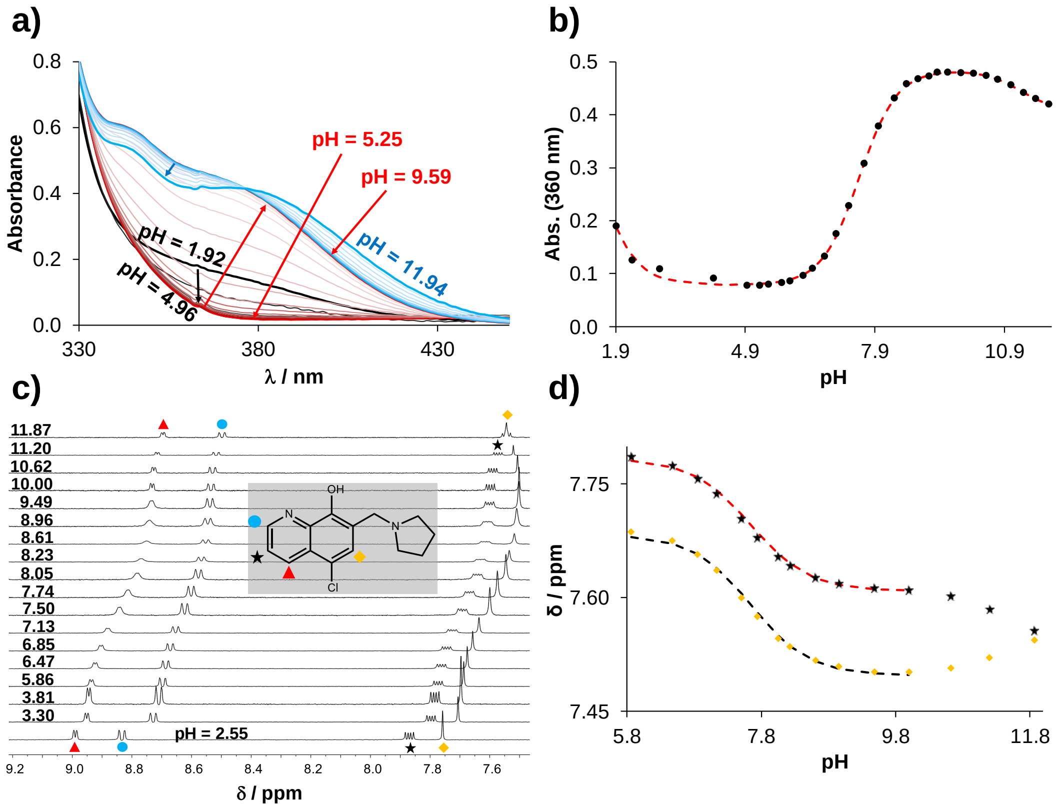

Figure S20. (a) UV-vis spectra of HQCl-pyr at increasing pH values and (b) absorbance values at 360 nm (●) plotted against pH along with the fitted (dashed) line. The titrations were conducted in the presence of 5 equiv. EDTA. {$c_{\text{HQCl-pyr}}$ = 81 μM; I = 0.20 M KNO_3; ℓ = 2 cm; T = 25.0 °C} (c) ^{1}H NMR spectra in the low-field region of HQCl-pyr at various pH values and (d) chemical shifts (* and ♦ (yellow) denotes C^{3}H and C^{6}H protons, respectively) as a function of pH along with the fitted (dashed) lines. The chemical shift values are only fitted only in the pH range 5.8 – 9.9 to determine pK_a (OH). {$c_{\text{HQCl-pyr}}$ = 400 μM; 10% (*v/v*) D_2O; I = 0.20 M KNO_3; T = 25.0 °C}

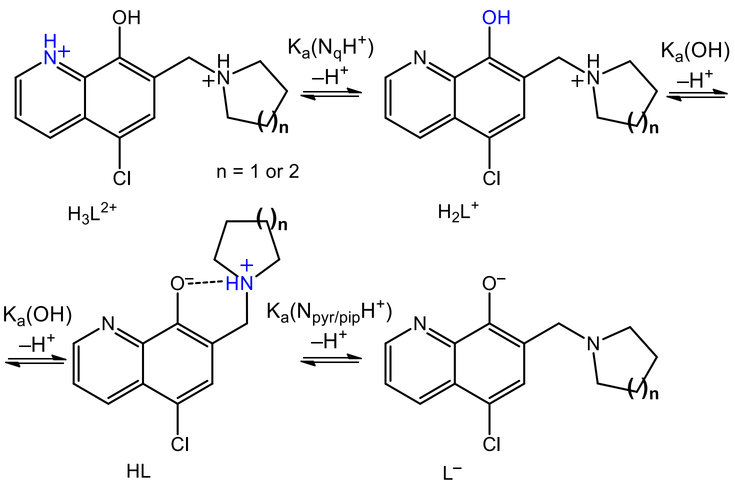

Scheme S2. Deprotonation processes of the two 8-hydroxyquinoline derived Mannich bases.

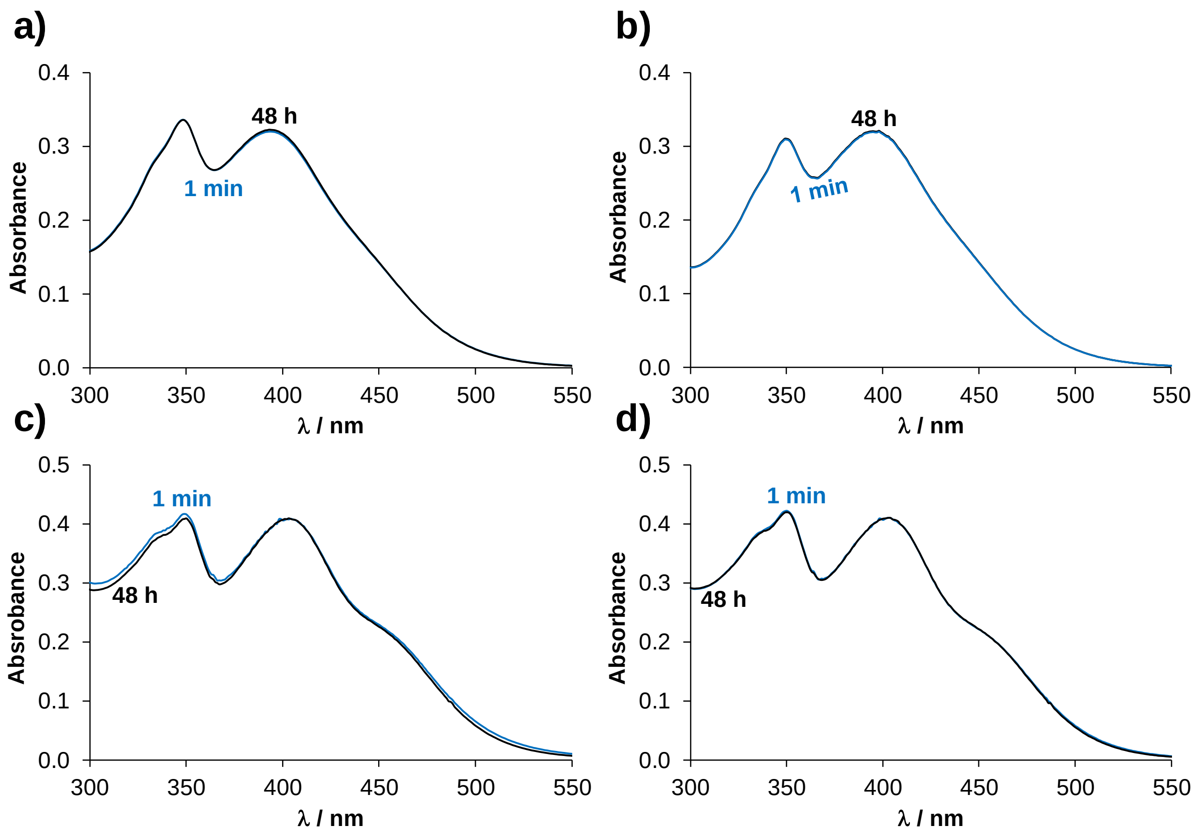

Figure S21. UV-vis spectra of the complexes a) (**1**), b) (**3**), c) (**2**) and d) (**4**) in PBS' recorded after 1 min after dissolution (blue solid lines) and after 48 h (black solid lines). {$c_{complex}$ = 100 μM; in PBS' (pH = 7.4); ℓ = 1 cm; T = 25.0 °C}

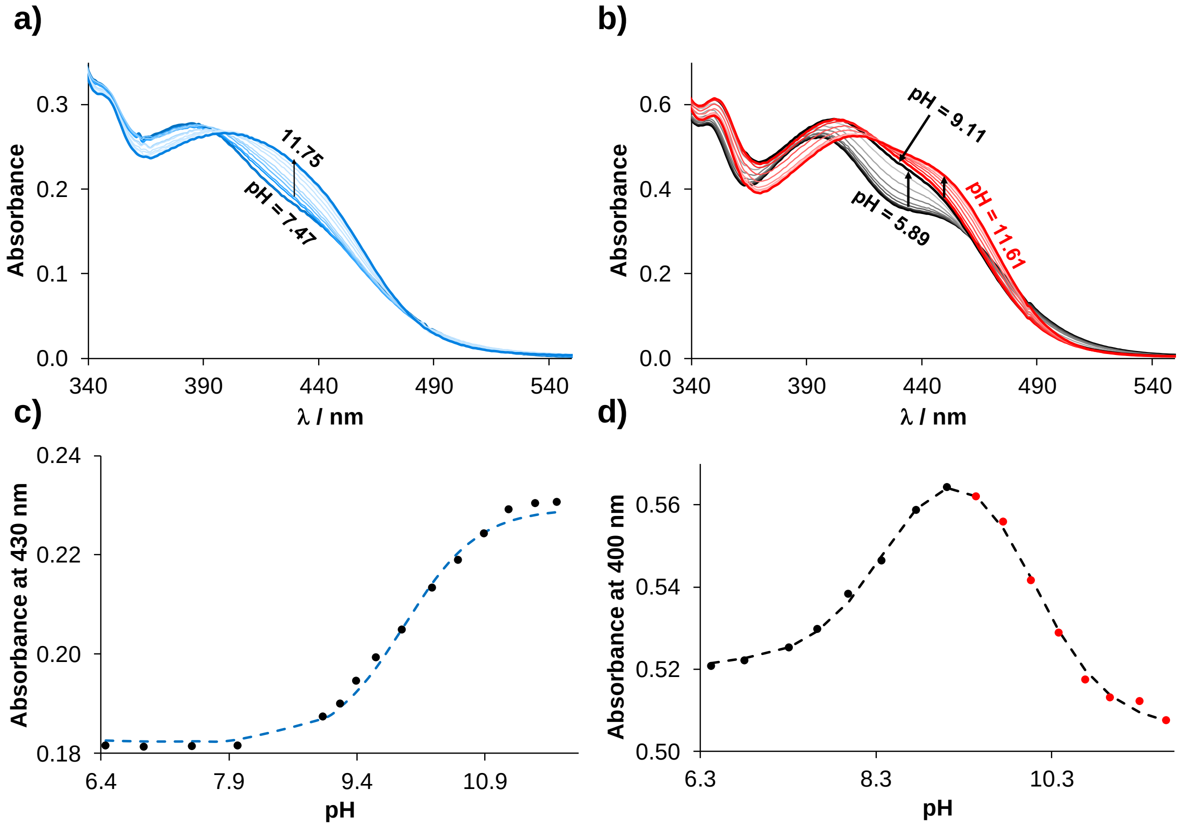

Figure S22. UV-vis spectra of (a) the RhCp* – HQCl-pyr and (b) the RuCym – HQCl-pip (1:1) systems at different pH values. (c) Absorbance values at 430 (● (black)) and (d) 400 nm (● (black) and ● (red)) plotted against pH with the fitted (dashed) lines for the same systems, respectively. {$c_{RhCp*} = c_{HQCl\text{-}pyr} = 36$ μM; $c_{RuCym} = c_{HQCl\text{-}pip} = 63$ μM; $I = 0.20$ M KNO_3; $\ell = 2$ cm; $T = 25.0$ °C}

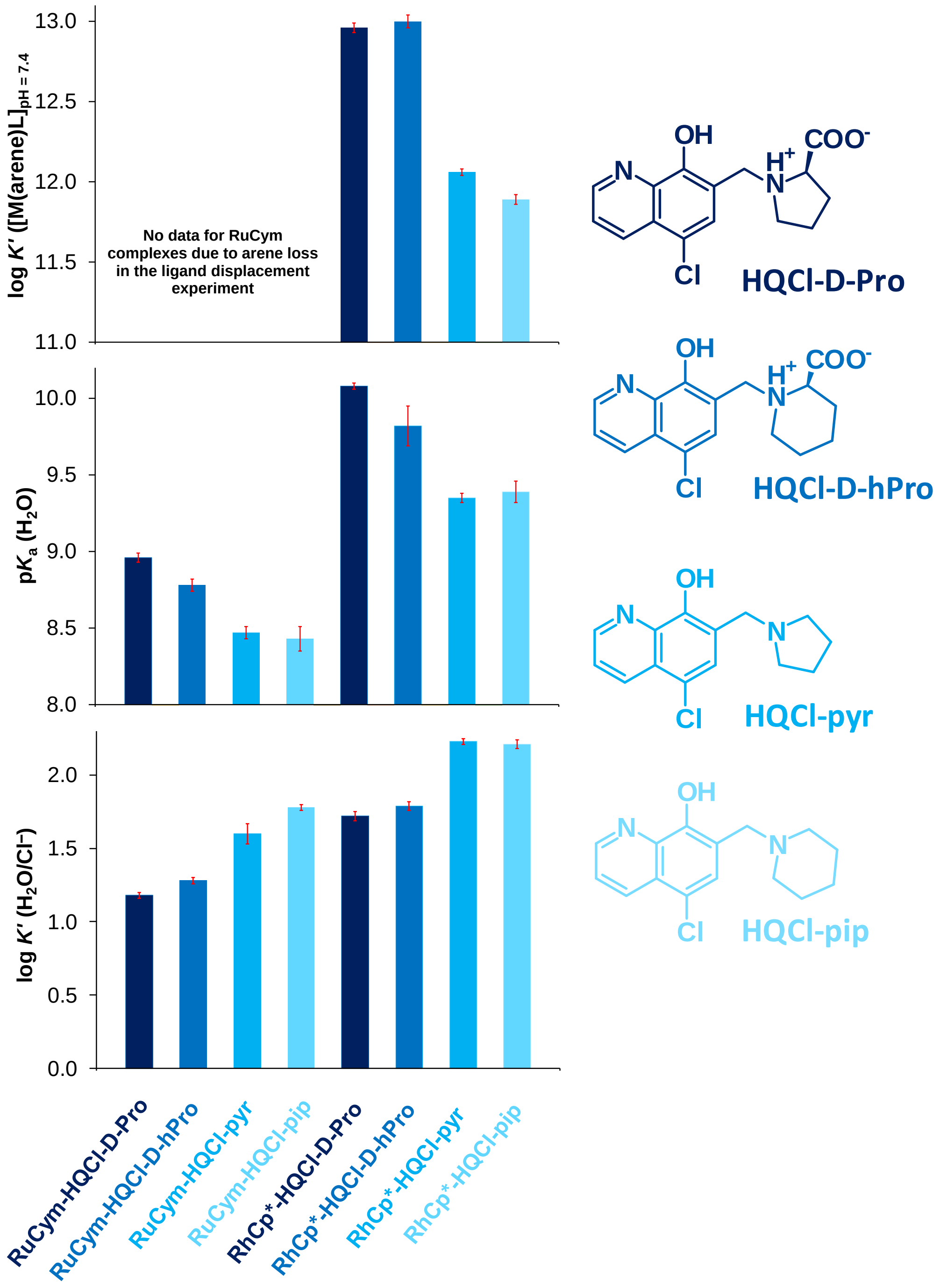

Figure S23. Conditional formation constants (logK' $[M(arene)L]_{7.4}$), pK_a (H_2O) and H_2O/Cl^- co-ligand exchange constants (logK' (H_2O/Cl^-) of RuCym and RhCp* complexes formed with HQCl-pyr, HQCl-pip, HQCl-D-Pro and HQCl-D-hPro. Data are taken for the two latter ligands from Ref. (SI1). {T = 25.0 °C}

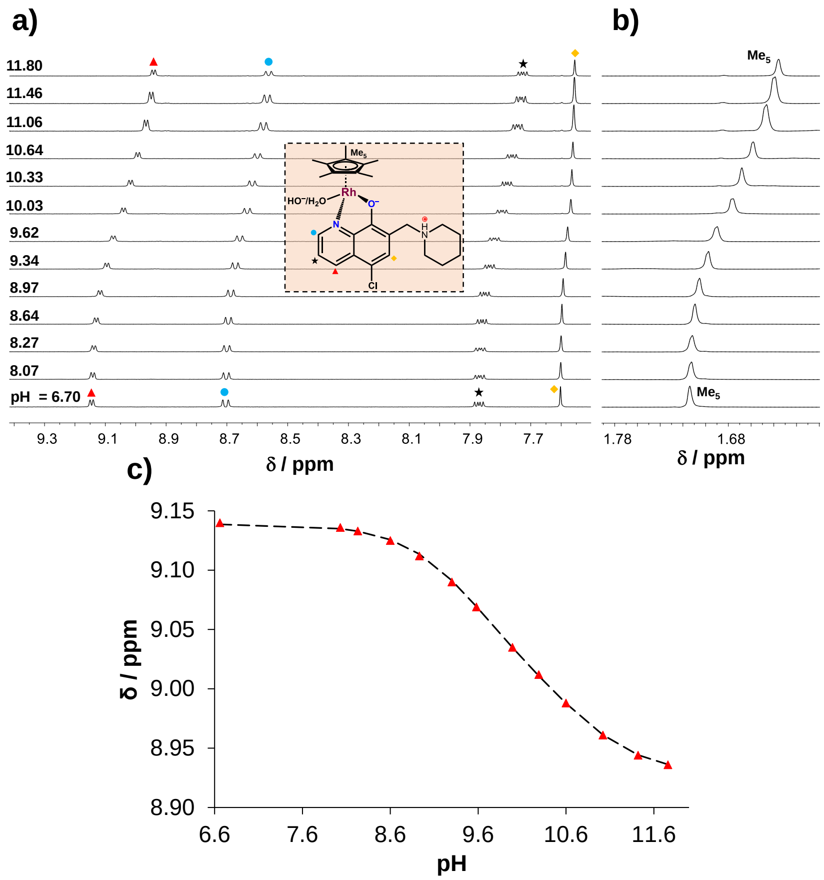

Figure S24. ^{1}H NMR spectra at the (a) down-field and (b) high-field region of the $[RhCp^*(H_2O)_3]^{2+}$ – HQCl-pip (1:1) system at various pH values (6.70 → 11.80). (c) Chemical shift values of C^4H proton (▲ (red)) with the fitted (dashed) line as a function of pH. From the spectral changes pK_a (H_2O) values could not be determined due to the strongly overlapping processes. {$c_{RhCp^*} = c_{ligand}$ = 1.47 mM, I = 0.20 M KNO_3, 10% (v/v) D_2O/H_2O, T = 25.0 °C}

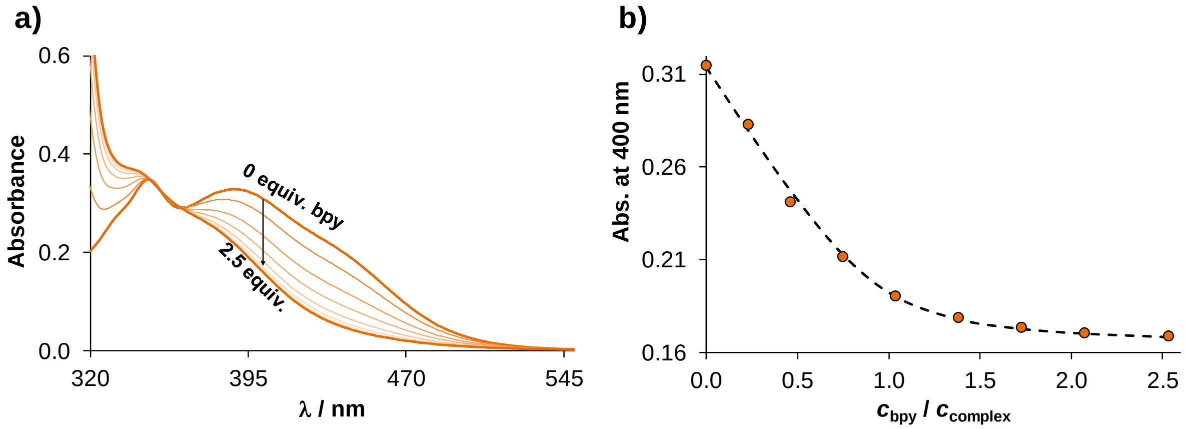

Figure S25. (a) UV-vis spectra of the RhCp* – HQCl-pyr (1:1) system in the absence and presence of various equivalents of 2,2'-bipyridine (bpy). (b) Absorbance values at 400 nm values in the function of c_{bpy} / $c_{complex}$ along with the fitted (dashed) line. {c_{RhCp*} = c_{ligand} = 72 μM; pH = 7.4 (20 mM phosphate buffer); I = 0.20 M KNO_3; ℓ = 1 cm; T = 25.0 °C}

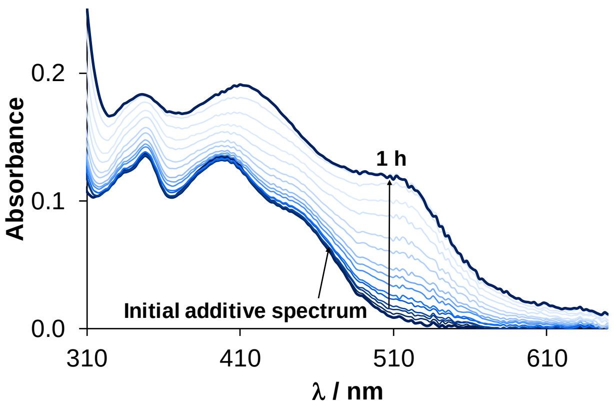

Figure S26. Time-dependent UV-vis spectra of the RuCym – HQCl-pyr (1:1) system in the absence and presence of 2 equivalents bpy. {$c_{complex}$ = 100 μM; c_{bpy} = 200 μM; pH = 7.4 (20 mM phosphate buffer); ℓ = 1 cm (tandem cuvette); I = 0.20 M KNO_3; T = 25.0 °C}

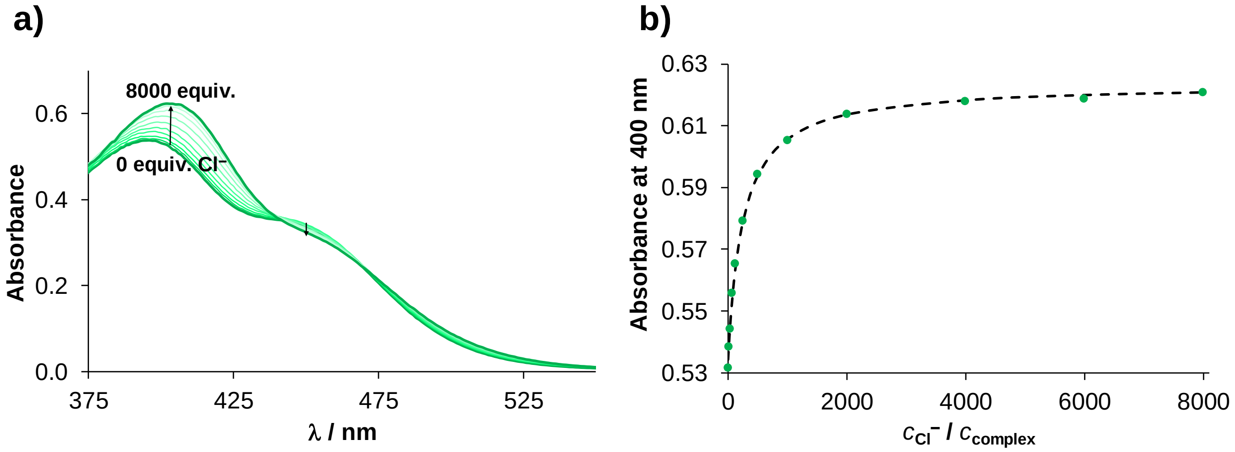

Figure S27. (a) UV-vis spectra of complex (**4**) in the absence and presence of various equivalents of chloride ions. (b) Absorbance values at 400 nm values in the function of c_{Cl-} / $c_{complex}$ along with the fitted (dashed) line. {$c_{complex}$ = 63 µM; pH = 6.0 (20 mM phosphate buffer); ℓ = 2 cm; *T* = 25.0 °C}

Table S4. Log$D_{7.4}$ values at pH = 7.4 of the studied complexes listed with the analogous PHQ complexes for comparison. {*I* = 0.20 M KNO_3; *T* = 25.0 °C}

**Compound**	**log$D_{7.4}$**		
	$c(Cl^-)$ = 4 mM	$c(Cl^-)$ = 24 mM	$c(Cl^-)$ = 100 mM
HQCl-pyr	–	–	+1.83 ± 0.02
HQCl-pip	–	–	+2.54 ± 0.01
PHQ [a]	–	–	+0.93
RhCp*–HQCl-pyr (**1**)	–0.11 ± 0.01	+0.12 ± 0.01	+0.22 ± 0.01
RhCp*–HQCl-pip (**3**)	+0.34 ± 0.01	+0.58 ± 0.01	+0.60 ± 0.01
RhCp*–PHQ [a]	–	–	–0.55
RuCym–HQCl-pyr (**2**)	–0.12 ± 0.02	+0.23 ± 0.03	+0.46 ± 0.02
RuCym–HQCl-pip (**4**)	+0.35 ± 0.02	+0.72 ± 0.02	+0.89 ± 0.02
RuCym–PHQ [a]	–	–	–0.78

[a] Data are taken from Ref. (SI2).

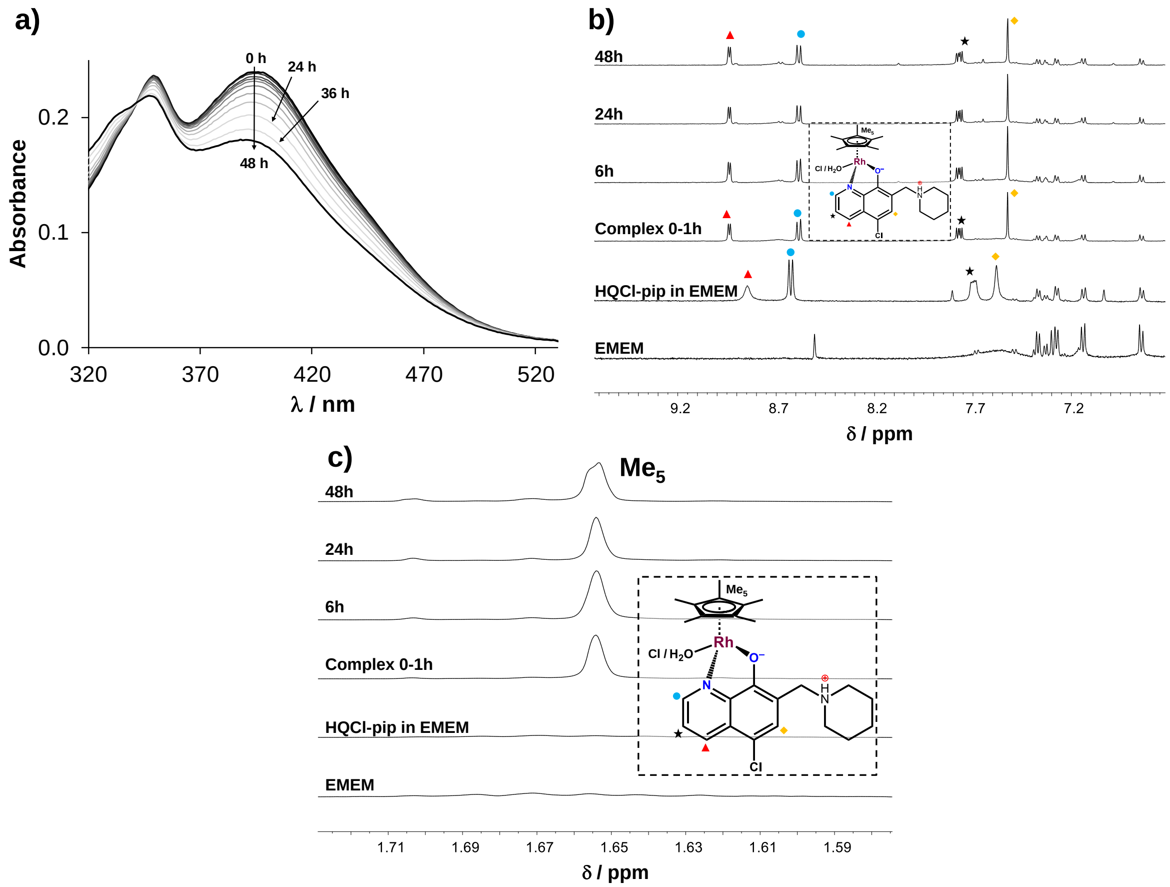

Figure S28. (a) UV-vis spectra of complex (**3**) in EMEM followed over time. {$c_{complex}$ = 50 µM; ℓ = 1 cm; T = 25.0 °C} ^{1}H NMR spectrum of EMEM, HQCl-pip and (**3**) in EMEM in the (b) low-field and (c) high-field region followed over time (0 – 48 h). {$c_{complex}$ = 1.5 mM; 10% (v/v) D_2O; T = 25.0 °C}

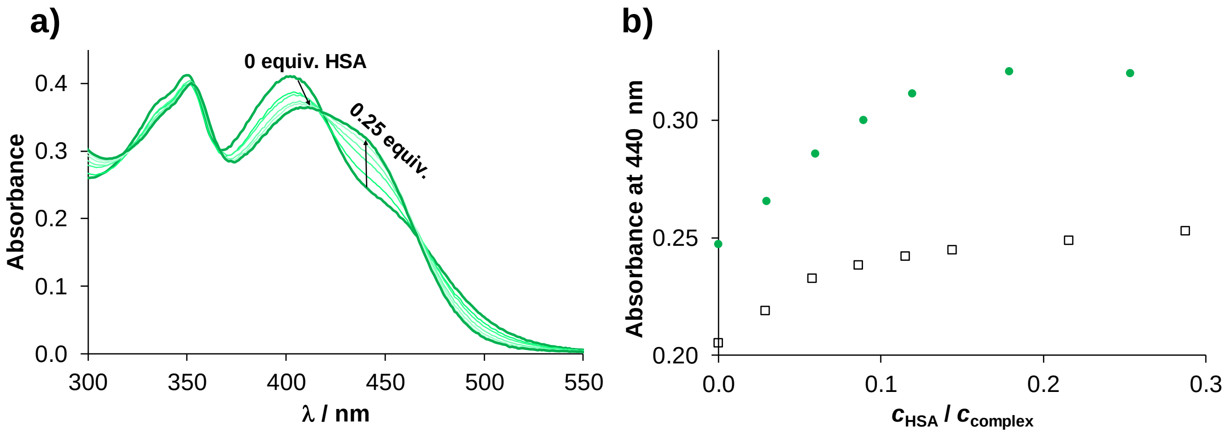

Figure S29. (a) UV-vis spectra of complex (**4**) in the absence and presence of various equivalents HSA. (b) Absorbance values at 440 nm in the function of c_{HSA} / $c_{complex}$ in the case of (**4**) (●) and (**3**) (□). {$c_{complex}$ = 92 or 100 µM; c_{HSA} = 0 – 37 µM; pH = 7.4 (PBS’); ℓ = 1 cm; T = 25.0 °C; waiting time: 24 h; waiting time: 24 h}

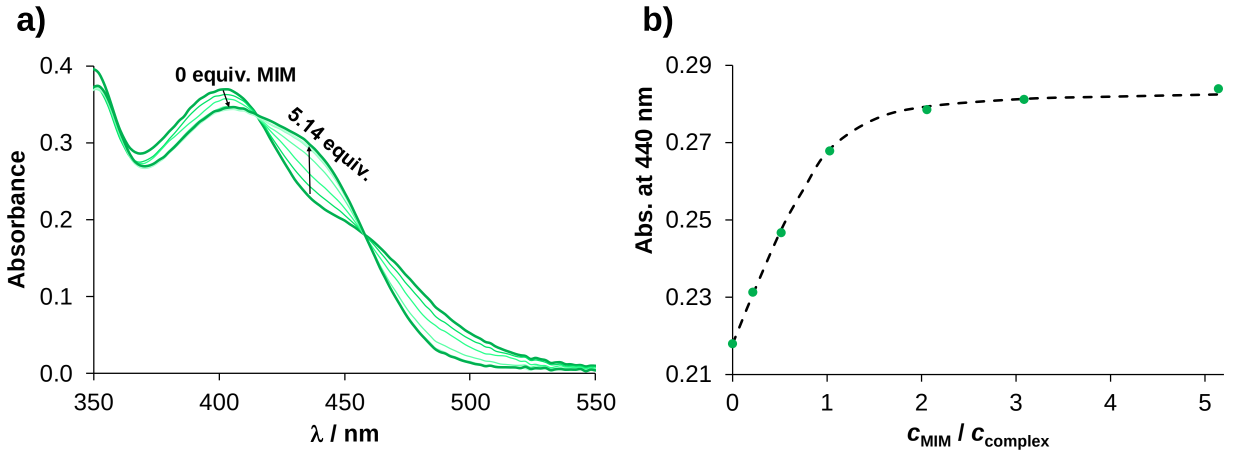

Figure S30. (a) UV-vis spectra of complex (**4**) in the absence and presence of various equivalents MIM. (b) Absorbance values at 440 nm in the function of c_{MIM} / $c_{complex}$ for the same complex (● (green)) along with the fitted (dashed) line. {$c_{complex}$ = 40 μM; c_{MIM} = 0 – 206 μM; pH = 7.4 (PBS'); ℓ = 2 cm; T = 25.0 °C; waiting time: 24 h}

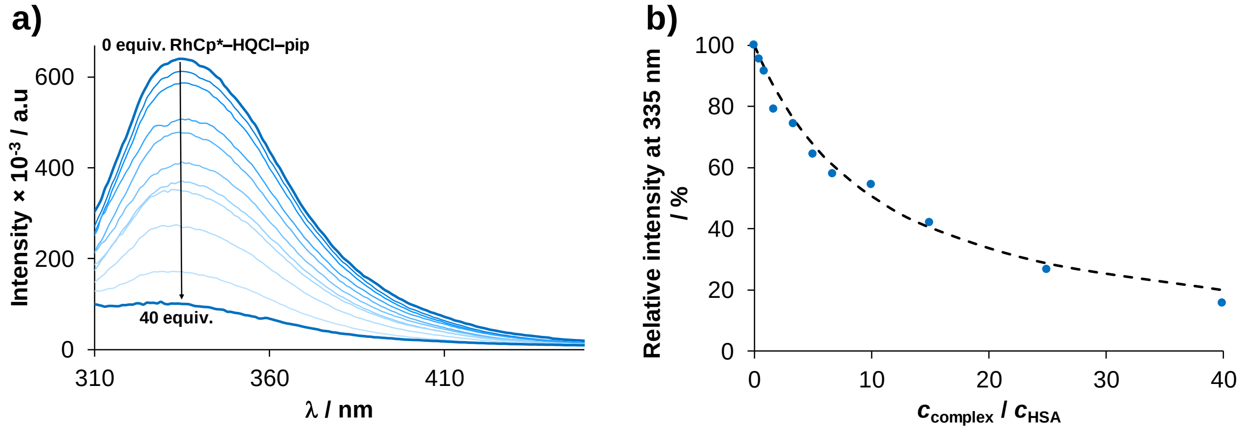

Figure S31. (a) Fluorescence emission spectra of HSA in the absence and presence of various amount of complex (**3**). (b) Experimental (● (blue)) and calculated (dashed line) relative intensities at 335 nm as a function of $c_{complex}$ / c_{HSA}. {c_{HSA} = 1 μM; $c_{compound}$ = 0 – 41 μM; λ_{EX} = 295 nm; pH = 7.4 (PBS' buffer); T = 25 °C; ℓ = 1 cm; waiting time: 24 h}

Table S5. *In vitro* cytotoxicity (IC_{50} in μM) of HQCl-pyr and HQCl-pip obtained in MES-SA, MES-SA/Dx5 (in absence and presence of 1 μM of the P-gp inhibitor tariquidar (TQ); A431 and A431-B1 (72 h incubation time) cell lines in monoculture, assessed with PrestoBlue viability assay.[SI3] Data are also shown for PHQ and its RhCp* and RuCym complexes for comparison.[SI2] IC_{50} values determined in the present work in Colo205 and Colo320 cells (72 h incubation time), where doxorubicin was used as a positive control.

**IC_{50} / μM**	**MES-SA**	**MES-SA/Dx5**	**SR**	**reference**
HQCl-pyr	3.46 ± 0.51	0.30 ± 0.06	11.53	(SI3)
HQCl-pip	2.49 ± 0.39	0.29 ± 0.05	8.71	(SI3)
PHQ	3.63 ± 0.58	0.62 ± 0.03	5.85	(SI2)
RhCp* complex of PHQ	4.63 ± 0.35	0.90 ± 0.12	5.14	(SI2)
RuCym complex of PHQ	5.25 ± 0.88	19.8 ± 5.4	0.27	(SI2)
HQCl-pyr + TQ	4.34 ± 0.45	3.21 ± 0.44	1.35	(SI3)
HQCl-pip + TQ	3.38 ± 0.36	2.58 ± 0.39	1.31	(SI3)
PHQ + TQ	3.46 ± 0.02	3.75 ± 0.51	0.92	(SI2)
RhCp* complex of PHQ + TQ	4.75 ± 0.07	4.41 ± 0.41	1.08	(SI2)
RuCym complex of PHQ + TQ	5.54 ± 0.70	66 ± 33	0.08	(SI2)
	**A431**	**A431-B1**	**SR**	
HQCl-pyr	7.45 ± 0.15	1.97 ± 0.25	3.79	(SI3)
HQCl-pip	6.67 ± 1.69	1.69 ± 0.27	3.96	(SI3)
PHQ	10.13 ± 2.95	2.48 ± 0.70	4.09	(SI3)
	**Colo205**	**Colo320**	**SR**	
HQCl-pyr	1.82 ± 0.23	0.91 ± 0.19	2.0	this work
HQCl-pip	1.05 ± 0.11	0.43 ± 0.03	2.4	this work
doxorubicin	0.05 ± 0.01	0.19 ± 0.02	0.3	this work

Table S6. Antibacterial effect (minimum inhibitory concentration (MIC) in μM) of the studied ligands and their complexes (**1** – **4**) obtained in the Gram-negative *Escherichia coli* and Gram-positive *Staphylococcus aureus* (methicillin susceptible and methicillin resistant (MRSA) strains.

**MIC / μM**	***E. coli*** **ATCC 25922**	***S. aureus*** **ATCC 25923**	***S. aureus*** **MRSA ATCC 43300**
HQCl-pyr	50	50	50
HQCl-pip	50	50	25
(**1**)	50	50	50
(**2**)	>100	>100	>100
(**3**)	50	50	25
(**4**)	>100	>100	>100

References

(SI1) Pivarcsik, T.; Dömötör, O.; Mészáros, J.P.; May, N.V.; Spengler, G.; Csuvik, O.; Szatmári, I.; Enyedy, É.A. 8-Hydroxyquinoline-Amino Acid Hybrids and Their Half-Sandwich Rh and Ru Complexes: Synthesis, Anticancer Activities, Solution Chemistry and Interaction with Biomolecules. *Int. J. Mol. Sci.* **2021**, *22*, 11281. https://doi.org/10.3390/ijms222011281

(SI2) Dömötör, O.; Pape, V.F.S.; May, N.V.; Szakács, G.; Enyedy, É.A. Comparative solution equilibrium studies of antitumor ruthenium(η^6-*p*-cymene) and rhodium(η^5-C_5Me_5) complexes of 8-hydroxyquinolines. *Dalton Trans.* **2017**, *46*, 4382 – 4396. https://doi.org/10.1039/C7DT00439G

(SI3) Pape, V.F.S.; Palkó, R.; Tóth, S.; Szabó, M.J.; Sessler, J.; Dormán, G. ; Enyedy, É.A.; Soós, T. ; Szatmári, I.; Szakács, G. Structure–Activity Relationships of 8-Hydroxyquinoline-Derived Mannich Bases with Tertiary Amines Targeting Multidrug-Resistant Cancer. *J. Med. Chem.* **2022**, *65*, 7729 – 7745. https://doi.org/10.1021/acs.jmedchem.2c00076
